# Remodeling of the cell membrane-associated protein pool affects adhesive membrane properties in filaggrin insufficient keratinocytes and impacts distinct cellular and organellar functions

**DOI:** 10.1186/s12915-025-02499-y

**Published:** 2026-01-08

**Authors:** Adrian Kobiela, Mikołaj Klimczuk, Paweł Kamil Serafin, Kamila Kitowska, Anna Biernacka, Reza Abouali, Jorge Bernardino de la Serna, Xinwen Wang, Felicja Gajdowska, Aniela Kosobucka, Aleksandra Małgorzata Siedlar, Amandine Hauer, Lilit Hovhannisyan, Aleksandra Bogucka, Jos P. H. Smits, Ellen H. van den Bogaard, Rafał Sądej, Graham S. Ogg, Danuta Gutowska-Owsiak

**Affiliations:** 1 Laboratory of Experimental and Translational Immunology, Intercollegiate Faculty of Biotechnology of the University of Gdańsk and the Medical University of Gdańsk, Gdańsk, Poland; 2 Medical Research Council Translational Immune Discovery Unit (MRC TIDU), Weatherall Institute of Molecular Medicine (WIMM), University of Oxford, Oxford, UK; 3 Laboratory of Cell Biology and Immunology, Institute of Medical Biotechnology and Experimental Oncology, Intercollegiate Faculty of Biotechnology of University of Gdańsk and Medical University of Gdańsk, Medical University of Gdańsk, Gdańsk, Poland; 4 Laboratory of Molecular Enzymology and Oncology, Institute of Medical Biotechnology and Experimental Oncology, Intercollegiate Faculty of Biotechnology University of Gdańsk and Medical University of Gdańsk, Medical University of Gdańsk, Gdańsk, Poland; 5 National Heart and Lung Institute, Imperial College London, Sir Alexander Fleming Building, London, UK; 6 Department of Oral Medicine, School of Stomatology, The Fourth Military Medical University, Xi’an, China; 7 Laboratory of Experimental and Translational Allergology and Pneumology, Medical University of Gdańsk, Gdańsk, Poland; 8 The Mass Spectrometry Laboratory, Intercollegiate Faculty of Biotechnology of University of Gdańsk and Medical University of Gdańsk, Gdańsk, Poland; 9 Department of Physiology, Medical University of Gdańsk, Gdańsk, Poland; 10 Department of Dermatology, Research Institute for Medical Innovation, Radboud University Medical Center (Radboudumc), Nijmegen, The Netherlands

**Keywords:** Keratinocyte, Filaggrin, Exosome, SEV, Membrane, Adhesion

## Abstract

**Background:**

Atopic dermatitis (AD) is a highly prevalent inflammatory skin disease, affecting up to 30% of children at some point in their life and frequently persisting into adulthood. Insufficiency in the late epidermal protein filaggrin is frequently observed in the lesional skin of patients, with direct and indirect impact on the skin barrier quality and function. We hypothesized that filaggrin reduction influences intracellular, surface, and derived extracellular membranes of keratinocytes with multiple impacts on the cell function.

**Results:**

Using filaggrin knockdown keratinocytes generated by shRNA interference (shFLG), we determined that the physical characteristics of the cellular membranes (reported by refractive index) are changed on a filaggrin insufficiency background. Using proteomics, protein binding modeling, and functional assays, we established that filaggrin insufficiency in keratinocytes results in changes in both organelles comprised of internal cellular membranes (i.e., small extracellular vesicles, sEVs) and the plasma membrane. We detected increased association of anti-adhesive proteins (tenascin-C and matrilin-2) with sEVs, resulting in a reduction of the fibronectin-1-mediated sEV uptake by dendritic cell subsets. At the same time, dysregulation of the tight junction and cell adhesion molecules at the level of the cell increased keratinocyte adhesiveness to reconstituted basement membrane substratum as well as faster gap closure in the wound healing assay. We also independently confirmed the findings on sEV uptake and wound healing in filaggrin knockout N/TERT-2G keratinocytes, which more closely resemble primary cells.

**Conclusions:**

We conclude that the alterations in different membrane compartments in filaggrin insufficiency are reflected in changes in keratinocyte functions of relevance to AD pathology, and strategies to target those could open up new therapeutic approaches.

**Supplementary Information:**

The online version contains supplementary material available at 10.1186/s12915-025-02499-y.

## Background

Atopic dermatitis (AD) is a common inflammatory skin disease in which both genetic and environmental factors play a role. The most significant inherited trait is represented by a genetic variant(s) in the gene encoding profilaggrin (*FLG*) [1]; a late epidermal barrier protein exerting multiple roles which are critical for skin barrier function. The reduction in filaggrin levels in the skin of AD patients may result from either the presence of a pathological *FLG* variant or/and the inflammatory process in the skin, as it is known that cytokines and other mediators such as histamine reduce its expression [2–10].

While filaggrin is a structural protein, it supports multiple barrier characteristics and is critical during keratinocyte differentiation, with the impact on some cellular processes and functions being seemingly unrelated cellular features [11–16]. This is most likely because of the complex effect on keratinocyte differentiation, which normally incorporates several coordinated processes activated during epidermal stratification and cornification. In addition, both the in vitro* models* and ex vivo studies show that filaggrin insufficiency results in immune dysregulation and overactivation of innate and adaptive immune pathways [17–22]. Importantly, dysbiosis, suggesting altered pathogen clearance, is often observed in the pathological *FLG* variant carriers [22, 23]; frequent *S. aureus* superinfections [24], enrichment in *Candida* and *Malassezia* species as well as fulminant infection with herpes simplex virus (known as *eczema herpeticum*) contribute to the clinical deterioration in patients. Genetic studies indicated that those *FLG* variants are also linked to the other manifestations of the “allergic march,” i.e., asthma, rhinitis, and food allergy, despite no expression of the protein physiologically in the affected organs, i.e., the lungs, suggesting the impact of the protein beyond the skin.

We have previously shown that filaggrin insufficiency contributes to allergic inflammation by affecting small extracellular vesicle (sEV)-mediated cellular communication from keratinocytes to immune cells [25]. This study also provided some suggestions on more extensive compartmental changes in keratinocytes; since we not only observed substantial differences in the lipid species’ contribution to the formation of sEV membranes but also found (by Gene Ontology analysis) that several cellular compartments are affected in this model.

In the current study, we hypothesized that organellar membranes may be broadly remodeled in the context of the suboptimal filaggrin levels in the cell and envisaged that those alterations may underpin altered cellular function(s), which may promote and shape AD hallmarks in patients.

## Results

### Alteration of physical membrane characteristics with filaggrin insufficiency impacts internal membrane compartments

**Fig. 1:**
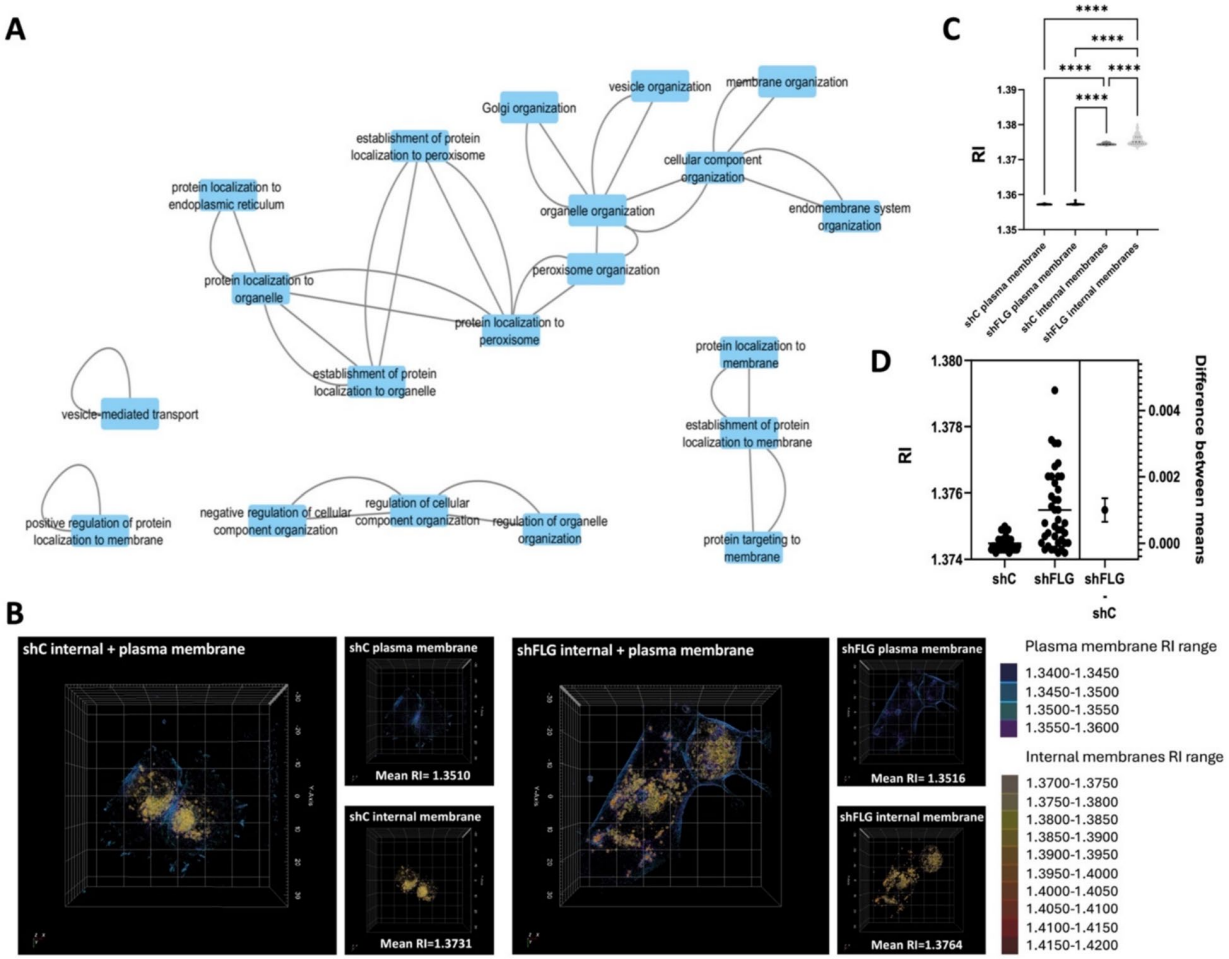
Filaggrin insufficiency alters physical characteristics of keratinocyte internal membranes.** A** GO terms network representing connections between the membrane-related GO terms identified as differential under the filaggrin insufficiency background; **B**–**D** 3D label-free holotomographic imaging analysis, showing refractive index (RI) of plasma membrane and internal membranes of shC and shFLG keratinocytes; **B** example images; x/y plane shown; RI of membranes is shown as a color gradient; **C** pooled RI data from *n* = 45 cells imaged per replicate; data representing two separate experiments; means and SEM are shown; Kruskal–Wallis test; ****p < 0.0001; **D** pooled internal membrane RI data from (C); *t*-test

Integration of Gene Ontology (GO) analysis carried out by us previously [25] on several datasets obtained from cultured keratinocytes, organotypic models, and AD skin samples indicated possible changes in the internal cellular membrane compartments (i.e., lysosomes, exosomes, membranes, endoplasmic reticulum, endoplasmic reticulum membrane) on a filaggrin insufficiency background. Specifically, GO or Reactome terms which we found significantly enriched included endomembrane system organization (GO:0010256), membrane organization (GO:0061024), or Membrane Trafficking (R-HSA-199991) as shown in Fig. 1A (full list of terms and their definitions can be found in Additional file 2: Table S1). Hence, such remodeling might have a significant functional impact on the aforementioned intracellular membrane compartments or organelles or the plasma membrane itself.

Hence, in this study, we set out to investigate filaggrin-insufficient keratinocytes in which filaggrin expression was reduced by shRNA interference (shFLG HaCaTs) and control shC cells. First, we compared the physical parameters, which report on the changes in the membrane quality. To this end, we assessed the membrane stiffness, which we previously used to study keratinocyte differentiation, by carrying out experiments with Laurdan [26]. Laurdan is a polarity-sensitive dye, which reports on a degree of lateral lipid packing in a membrane by quantifying general polarization (GP) function [27]. These experiments did not demonstrate any differences in lipid packing between shC and shFLG cells when testing in the plate reader-assisted high-throughput assay [26] (Additional file 1: Fig. S1). However, a whole-cell population-based study may not reflect the subtle differences at the level of a single cell. It may average lipid packing values obtained at the plasma membrane with the values from internal organelles, as we previously showed [26]. To discern better higher density by membranous compartments at the single-cell level, we next turned to holotomographic microscopy and measured membrane refractive index (RI) at the plasma membrane and internal organelles. RI is a measurement of the optical density, and its differences are directly correlated to condensation, i.e., denser cellular compartments and organelles yield a higher RI; however, unlike Laurdan, RI reports on both the lipid and protein content in a membrane. Here, we observed that RI values at the plasma membrane were similar in shC and shFLG cells. However, RI was significantly higher for the internal membranes than for plasma membranes, as shown in the RI color gradient images (Fig. 1B–D); moreover, we observed a further increase in the average RI of the internal membranes in shFLG in comparison to those in shC cells (Fig. 1D). To this end, while the difference between the means was not very pronounced, we noticed a very different spread of the RI values obtained for the internal membranes in the shFLG cells, suggesting much higher heterogeneity in their internal membrane composition. N.B.: The spread of the data points visible for the shFLG cells in Fig. 1C–D may indicate the difference that depends on the level of the sh-mediated filaggrin reduction, given that the pool of shFLG cells is heterogeneous, representing a very mixed population with different degrees of filaggrin knockdown.

### sEVs secreted on the filaggrin insufficiency background are enriched in anti-adhesive proteins

**Fig. 2:**
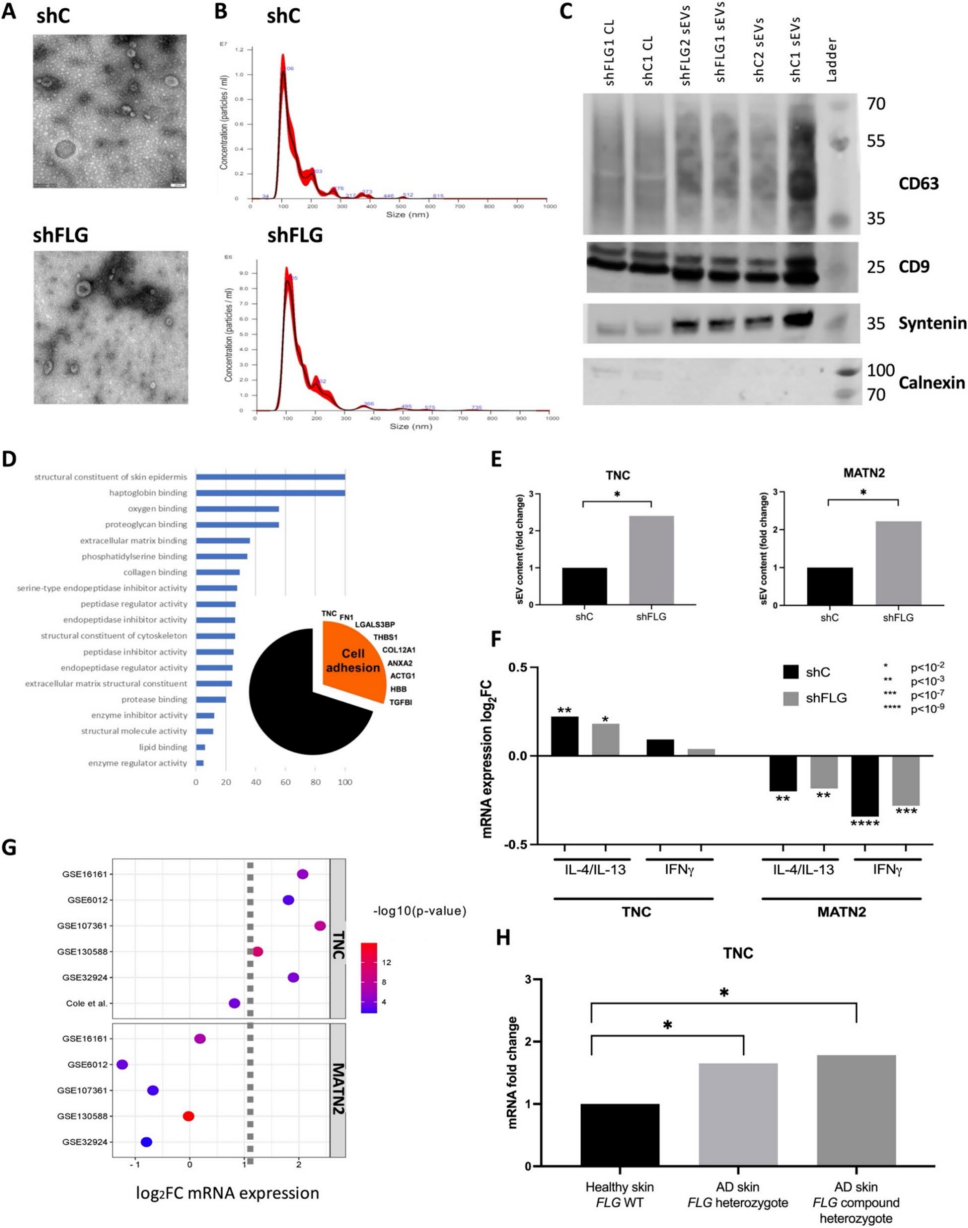
Anti-adhesive proteins enriched in shFLG_sEVs_ are differentially regulated by AD cytokines and in AD skin. **A** Electron microscopy images of keratinocyte-derived sEVs. **B** Size profiles of keratinocyte-derived sEVs by Nanoparticle Tracking Analysis (NTA). **C** Western blot showing enrichment of exosomal markers in keratinocyte-derived sEVs from *n* = 2 biological replicates. **D** Enrichment of Gene Ontology (GO) terms for molecular function in keratinocyte sEV proteins; pie chart inset showing sEV proteins to which the “Cell adhesion” GO term is assigned. **E** Differences in TNC and MATN2 abundance between shC_sEV_ and shFLG_sEV_; pooled data from *n* = 4 replicates; unpaired *t*-test with FDR-adjusted *p*-values; *p < 0.05, **p < 0.01. **F** Expression of TNC and MATN2 mRNA in shC and shFLG keratinocytes under stimulation with type 2 cytokines and IFNγ; pooled data from *n* = 3 biological replicates; *t*-test; *p < 0.01, **p < 0.001, ***p < 10^–7^, ****p < 10^–9^. **G** Bubble plot showing TNC and MATN2 mRNA expression in the skin of AD patients; data obtained from publicly available data sets via GEO2R and a study by Cole et al. [31]; mRNA expression between non-lesional AD vs healthy controls skin for Cole et al. [31] and lesional AD vs healthy controls skin for the remaining datasets was compared; mRNA expression changes for GEO2R-analyzed samples were calculated using default settings; FDR-adjusted *p*-values are reported; number of patients/healthy controls per dataset: GSE16161, 9 AD lesional (AD-L) and 9 healthy controls (HC); GSE6012, 10 AD-L and 10 HC; GSE107361, 39 AD-L and 29 HC; GSE130588, 56 AD-L and 20 HC; GSE32924, 13 AD-L and 8 HC; Cole et al. [31], 7 AD non-lesional *FLG* compound heterozygotes and 8 healthy *FLG* WT. **H** TNC mRNA expression data in non-lesional skin of AD patients with different *FLG* status compared to healthy controls from Cole et al. [31]; data from 8 *FLG* WT healthy subjects, 12 *FLG* heterozygous AD patients, and 7 *FLG* compound heterozygous patients is shown; FDR-adjusted *p*-values shown; *p < 0.05

Since our results so far confirmed that intracellular membranes are altered on the filaggrin insufficiency background, we set out to investigate secreted exosome-enriched small extracellular vesicles (sEVs), as an example membrane-bound organelle of intracellular origin. We have previously described differences in the lipid content between shC_sEV_ and shFLG_sEV_ and showed how those differences may affect adaptive immune responses and contribute to allergic inflammation in AD [25]. To test whether the sEV protein compartment, i.e., proteins either incorporated into the sEV membranes, membrane-associated, or intraluminal, are similarly altered on a filaggrin insufficiency background, we isolated sEVs from keratinocyte cultures and confirmed the exosomal enrichment by the morphology by transmission electron microscopy (TEM, characteristic cup shape of the vesicles), size profile by Nanoparticle Tracking Analysis (NTA, expected size around 100 nm), and WB analysis of the exosomal markers (positive markers: CD63, CD9, syntenin; negative marker: calnexin) (Fig. 2A–C; Additional file 1: Fig. S2, Additional file 4). Of note, while we did not observe any significant differences in the previous study [25] as far as the size distribution or TEM-assessed morphology is concerned, there could be a difference in their proteome. Hence, we next performed protein mass spectrometry of the sEVs and compared the results with the cellular proteome (Additional file 1: Fig. S3A–B). To this end, we observed that 12/30 sEV proteins were detected in whole keratinocyte lysates, in line with the expected enrichment of such proteins in the sEV cargo and in agreement with the notion that loading of the cargo into exosomes is a selective process rather than random sampling of the cellular content. At the same time, nearly all the differentially expressed proteins in shFLG keratinocytes have been previously identified in exosomes according to Vesiclepedia, a database of EV content, suggesting a potential broad impact of filaggrin insufficiency in cells on the composition and functionality of small vesicles (Additional file 1: Fig. S3C).

As for the proteins identified in the sEV fractions, we observed structural keratinocyte proteins (keratins), exosomal marker Heat-shock protein 70 protein 8 (HSPA8), and enzymes with roles in proteolytic degradation (e.g., proteasome subunits) across all samples (Additional file 1, Fig. S3D; Additional file 3: Table S2). Two of those were found in the top 50 differently abundant proteins in shFLG vs. shC (cellular) proteome (Additional file 1: Fig. S4), i.e., keratin-14 and galactin-3-binding protein (LGALS3BP).

GO analysis of sEV proteome demonstrated enrichment for proteins with a capacity for adhesion and binding (Fig. 2D), i.e., 9 of the total 30 proteins present in sEVs (Fig. 2D, inset). Here, several proteins that play a role in cell adhesion are linked to the extracellular matrix; these seem to create a protein network with a central importance of fibronectin-1 (FN1) (Additional file 1: Fig. S3E). These proteins are likely associated with the vesicles as a protein component of the sEV corona rather than included in their structure, which hints at the possibility that FN1 or proteins that are members of its network may play an important role in mediating interactions between sEVs and various cellular components, including the plasma membrane.

The comparison between shC_sEV_ and shFLG_sEV_ showed a significant increase in the abundance of tenascin- C (TNC) and matrilin-2 (MATN2) in sEVs from keratinocytes in which filaggrin expression was knocked down (Fig. 2E). An increase in protein abundance, not compensated by any detectable decrease of other proteins, is in line with the RI results indicating a higher density of the internal membranes in the shFLG cells. At the same time, neither TNC nor MATN2 seems to be differentially regulated at the cell level since we observed similar expression at both mRNA [28] and protein levels in the shC and shFLG keratinocytes [25].

### Tenascin-C and matrilin-2 are differentially regulated by AD cytokines in AD skin

TNC is known to be upregulated in human primary keratinocytes stimulated with proinflammatory cytokines [29, 30]. Interestingly, when we compared the expression of TNC and MATN2 in keratinocytes upon exposure to AD-relevant cytokines, we found these proteins to be regulated by cytokines present in acute and chronic lesions (IL-4/IL-13 vs. IFNγ, respectively), regardless of the filaggrin status of the cells (Fig. 2F). Specifically, we observed that while the expression of TNC is increased by IL-4/IL-13, it is not affected by IFNγ, while MATN2 was significantly downregulated in both cases. In agreement with those data, we also identified studies that reported on those proteins in AD skin samples; the majority has shown TNC upregulation and MATN2 downregulation (Fig. 2G) [31], which may suggest the differential roles of those proteins during AD progression in the skin. Moreover, we found that the filaggrin status of AD patients impacts TNC expression in the skin (Fig. 2H). Specifically, data from Cole et al. [31] show increased TNC expression in the skin of AD *FLG* heterozygote or compound heterozygote patients in comparison to healthy *FLG* wild type controls. TNC dysregulation was not observed in AD wild type *FLG* subjects. NB: since the analyzed dataset did not report on the fold changes for genes that were not significantly up-/downregulated vs. Healthy group, the AD wild type group is not shown in the graph.

### Tenascin-C and matrilin-2 are included in the fibronectin-1 network and may bind this protein directly

**Fig. 3:**
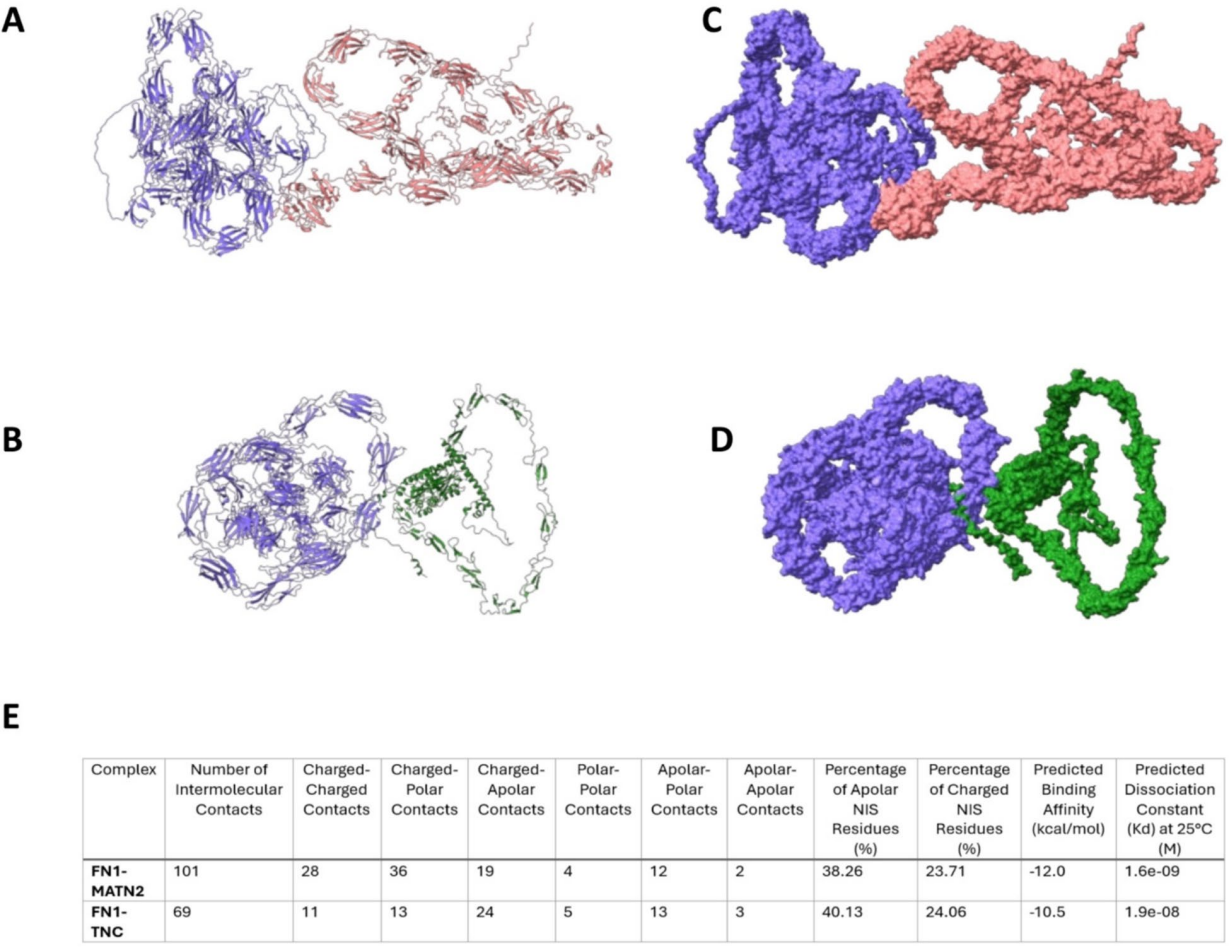
Tenascin-C and matrilin-2 are predicted to bind fibronectin-1. **A**–**D** Visual representations of the docked complexes of FN1 (purple) with TNC (pink) and MATN2 (green); ribbon models of the **A** FN1-TNC and **B** FN1-MATN2 complexes highlighting the overall structural interactions; surface models of **C** FN1-TNC and **D** FN1-MATN2 illustrating the interaction interfaces; binding prediction performed using the ChimeraX molecular visualization program. **E** Binding data for FN1 complexes generated using the PRODIGY web server

We next investigated the interactions of these proteins and found that both belong to the molecular network of FN1 (Additional file 1: Fig. S3F), which we previously recognized as a central component with adhesive functionality in the sEV fractions (Additional file 1: Fig. S3D; Table S2). Since it has been previously shown in chickens that TNC may interact directly with FN1, thereby blocking the FN1 domain FNIII13 [32] and exerting an anti-adhesive effect, we next investigated if the human TNC and MATN2 may also bind FN1. To this end, we carried out the analysis of the 3D protein structures obtained from the AlphaFold Protein Structure Database. Using ClusPro, we generated multiple docked models for the FN1-MATN2 and FN1-TNC interactions. From these, the models from Cluster 0 were selected for further analysis due to their favorable interaction energies. The representative weighted score obtained for FN1-MATN2 was − 353.4, while the score for FN1-TNC was − 290.4. Next, the selected models were visualized using UCSF ChimeraX to generate images for further analysis (Fig. 3A–D, Additional file 1: Fig. S5, Additional files 5–10, Movies S1–S6). These visualizations provided clear representations of the docked complexes, allowing for comparison between the FN1-MATN2 and FN1-TNC interactions. Notably, the relative positions of MATN2 and TNC when bound to FN1 suggest that they do not overlap and do not appear to compete for the same binding site.

Binding affinity calculations using PRODIGY further supported this observation. The FN1-MATN2 complex exhibited a predicted binding affinity of − 12.0 kcal/mol and a dissociation constant (Kd) of 1.6 − 09 M at 25 °C (Fig. 3E). In contrast, the FN1-TNC complex had a binding affinity of − 10.5 kcal/mol with a Kd of 1.9e − 08 M at 25 °C. These results indicate that FN1 has a higher binding affinity for MATN2 than for TNC, based on both the docking energy scores and the PRODIGY binding affinity predictions.

### sEVs secreted on filaggrin-insufficient background have reduced propensity to undergo DC uptake

**Fig. 4:**
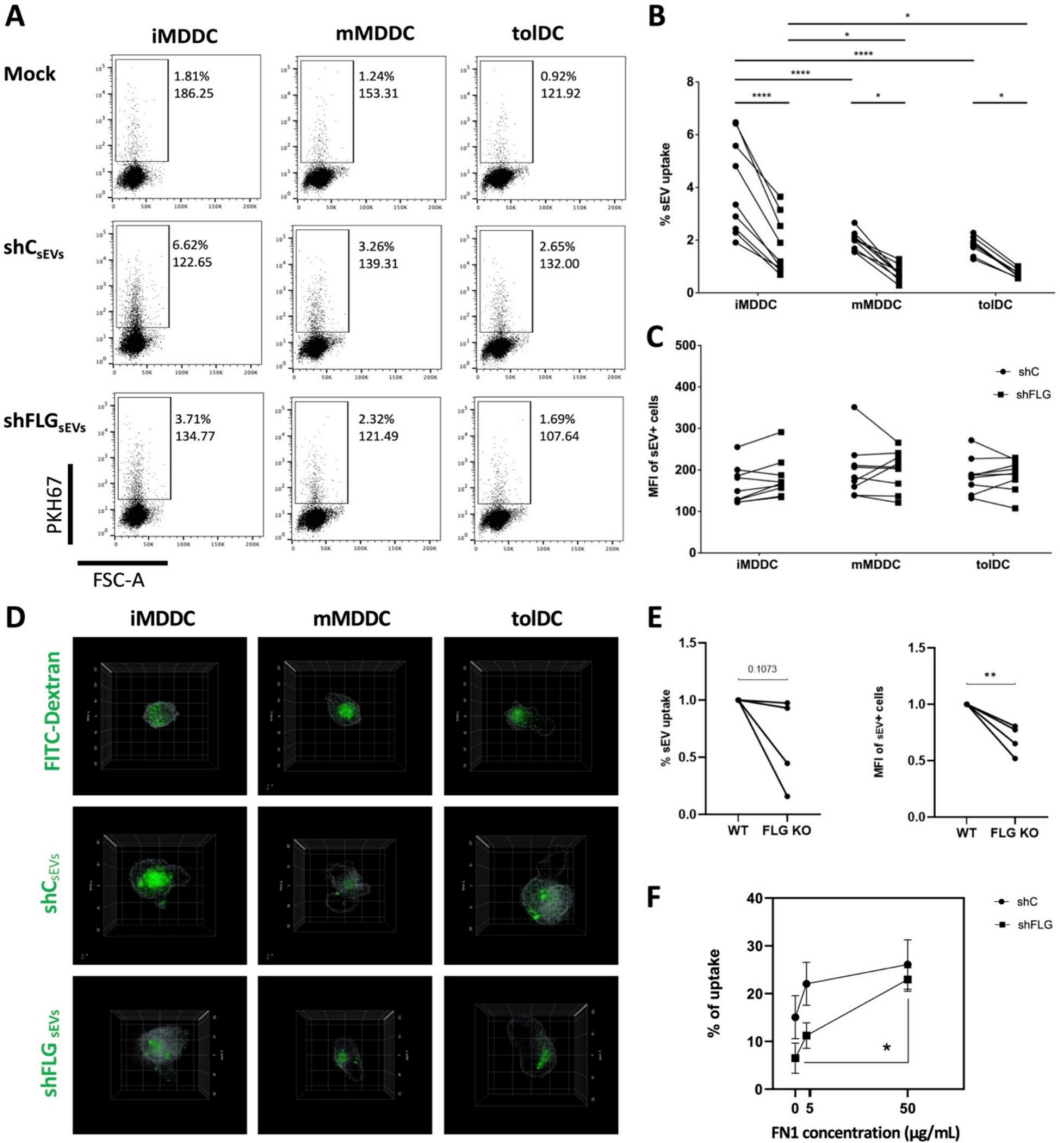
Cellular uptake of sEVs from shFLG is reduced in comparison to shC. **A**–**C** Uptake of density gradient-purified PKH67-labeled keratinocyte sEVs by MDDCs measured by flow cytometry; **A** example plots with % positive cells (upper values) and MFI (lower values) and summary of **B** % positive cells and** C** MFI; pooled data from *n* = 9 donors; means with SEM are shown; one-way ANOVA with Holm-Šídák’s multiple comparisons test; *p < 0.05, ****p < 0.0001. **D** 3D fluorescent holotomographic reconstruction images of the uptake of PKH67-labeled sEVs and FITC-conjugated dextran by MDDCs; example images of x/y planes; sEVs or fluorescent dextran in green. **E** Uptake of PKH67-labeled sEVs produced by N/TERT-2G by THP-1 cells; normalized data from *n* = 5 biological replicates; data normalized to uptake of WT sEVs = 1; unpaired *t*-test; **p < 0.01. **F** flow cytometry analysis of % uptake of FN1-coated and PKH67-labeled sEVs by the THP-1 model cell line; pooled data from *n* = 3 biological replicates; 2-way ANOVA, mean and SEM are represented; *p < 0.05

Since our results so far suggested that TNC and MATN2 in shFLG_sEV_ may act as anti-adhesive in humans, by affecting the FN1-mediated adhesion of sEVs to the recipient cell, we further investigated if there are any noticeable differences in sEV propensity to undergo cellular uptake. Such a difference could potentially impact the innate and adaptive immune responses in the skin, by influencing the delivery of antigens and innate mediators from environment-exposed keratinocytes to dendritic cells in the skin. To this end, we generated immature, mature, and tolerogenic monocyte-derived dendritic cells (iMDDCs, mMDDCs, and tolDCs, respectively; Additional file 1: Fig. S6A–B). Our tolDC model showed a decreased expression of co-stimulatory receptors CD40 and CD86 compared to mMDDCs while HLA-DR and CD1a levels were not affected; this is in line with several published tolDC models [33, 34]. We observed expected differences in the dextran uptake based on the DC functionality (the highest for iMDDCs, the lowest for tolDCs; Additional file 1: Fig. S6C–E), confirming the correctness of our models. We next pulsed the cells with density gradient-purified sEVs labeled with a membrane dye PKH67 and examined sEV uptake by flow cytometry and holotomography; we also included the mock control (staining performed without the addition of sEVs) for the former. The uptake of labeled sEVs by the DC subsets reflected the functionality of the cells, mirroring dextran uptake results (Fig. 4). As for the differences between the sEV sources, we observed a significant reduction of the shFLG_sEV_ uptake in comparison to the uptake of shC_sEV_ for all the DC models studied, both by flow cytometry (Fig. 4A–C) and holotomography with fluorescent imaging (Fig. 4D and zoom-ins in Additional file 1, Fig. S6F).

Since HaCaT keratinocytes may not represent primary keratinocytes closely, especially with their inability to fully differentiate into 3D models in vitro, indicating a defect in terminal differentiation, we also confirmed the findings with a CRISPR-mediated filaggrin knockout on the N/TERT-2G background [16]. The N/TERT-1/2 lines obtained in the Rheinwald laboratory, which can form a fully functional *stratum corneum*, are believed to be a model which better represents primary epidermal keratinocytes [35, 36]. We confirmed the presence of positive exosomal markers CD63, CD9, and Alix and the absence of negative markers calnexin and ApoA in ΔFLG and WT N/TERT-2G-derived sEVs by WB, as well as obtained size profiles of those vesicles by NTA (Additional file 1: Fig. S7A, Additional file 4); no differences between the two sEV populations were observed. Further results for these WT and ΔFLG lines also showed a reduced uptake of the sEVs generated on the filaggrin deficiency background by THP-1 cells (Fig. 4E and S7B); this reduction was especially highly significant for the MFI readout; however, a similar trend was also observed in the percentage of cells that internalized the sEVs. While such discrepancies may stem from the use of different cell types (MDDCs vs. THP-1) that received the sEVs, we could still confirm the negative impact of filaggrin insufficiency/deficiency in keratinocytes on the uptake of their sEVs by antigen-presenting cells using two keratinocyte cell line models.

Given that this differential sEV uptake was likely a consequence of the masking of the FN1 binding sites by accumulated TNC and MATN2, we next performed a “rescue experiment.” To this end, we preincubated shC_sEV_ and shFLG_sEV_ with recombinant human FN1, washed the unbound protein by ultracentrifugation, and performed uptake experiments with a model THP-1 cell line. The results showed an increase in the uptake of both shC_sEV_ and shFLG_sEV_ upon FN1 preincubation, and a significantly greater difference was observed for shFLG_sEV_ (Fig. 4F), suggesting that saturating FN1 reduces the differences resulting from the increased abundance of TNC and MATN2 and compensates for their anti-adhesive properties.

### Filaggrin insufficiency results in dysregulated expression of membrane proteins, leading to changes in cellular adhesiveness and the speed of wound closure by keratinocytes

**Fig. 5:**
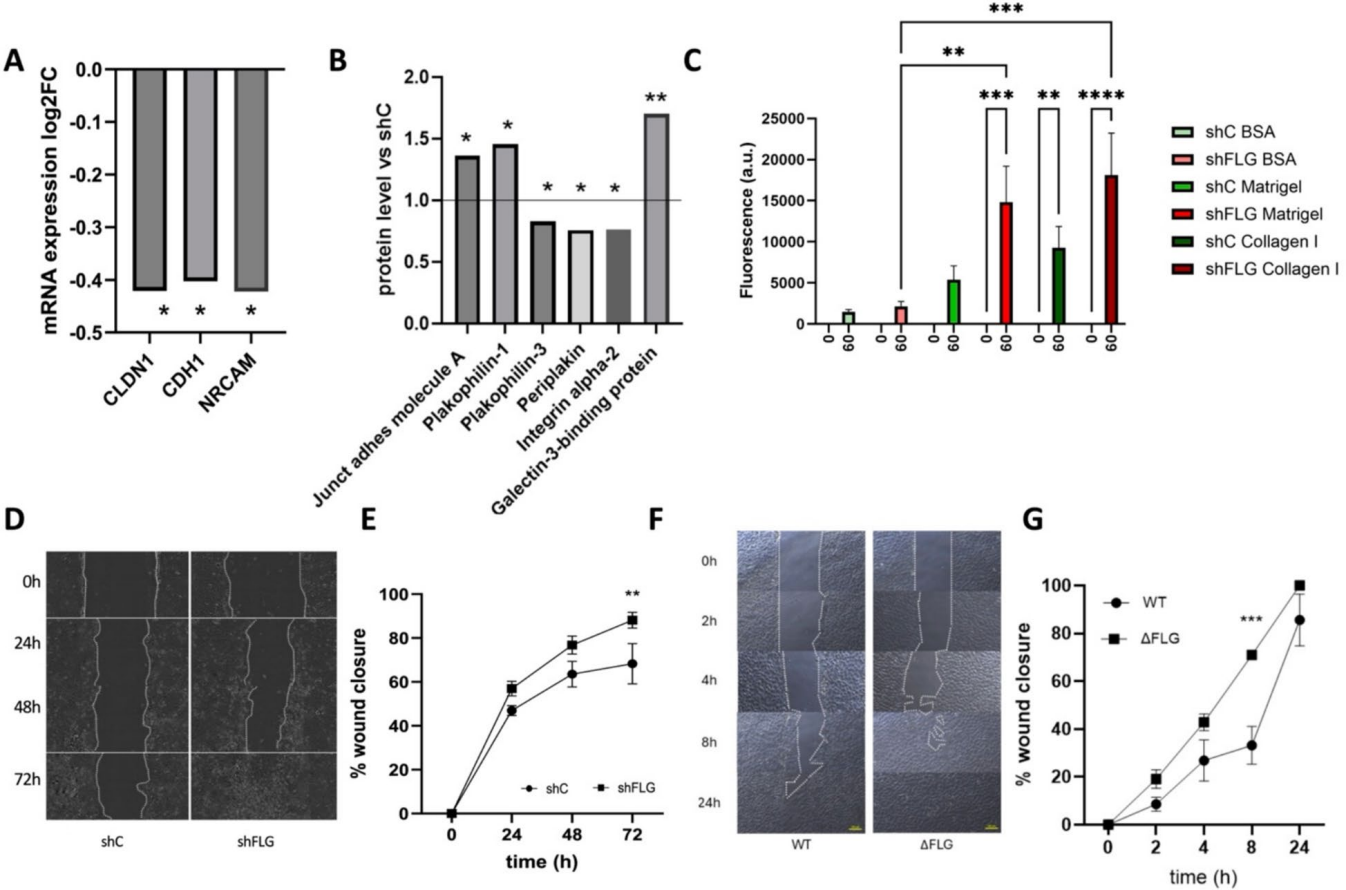
Filaggrin insufficiency alters adhesive and migratory properties of keratinocytes. **A**–**B** Expression of adhesion and junctional proteins by shFLG keratinocytes compared to shC at the **A** mRNA and **B** protein level; **A**, *n* = 3 biological replicates; *t*-test; **B**, *n* = 4 biological replicates; unpaired *t*-test; FDR-adjusted *p*-values are shown; *p < 0.05, ***p < 0.001. **C** Adhesion of shC and shFLG keratinocytes to cell culture plate wells coated with BSA (control), Matrigel, and collagen I; means with SEM are shown; *n* = 3 biological replicates; 2-way ANOVA; *p < 0.05. **D**–**E** Wound healing assay in shC and shFLG keratinocytes; **D** wound assay—example light microscopy images; **E** pooled data on a reduction in wound bed surface in time; *n* = 3 biological replicates; two-way ANOVA; **p < 0.01. **F**–**G** Wound healing assay in FLG WT and ΔFLG N/TERT-2G; **F** example light microscopy images; **G** pooled data showing reduction in wound size over time; *n* = 3 biological replicates; two-way ANOVA; ***p < 0.001

Finally, since our example organelles, shFLG_sEV_ showed reduced adhesion and uptake, we were also interested in testing if adhesion-related properties of the plasma membrane were also affected. To this end, we started by investigating the expression pattern of adhesion and junctional molecules, at both the mRNA and protein levels in the datasets we previously published (GEO repository identifier: GSE203409 [25, 28, 37] and Proteome Xchange identifier PXD026859 [25, 38], respectively). We noticed that both datasets reported on a different set of relevant and significantly regulated genes/proteins (Fig. 5A–B) and found that several involved in cell–cell and cell-extracellular matrix adhesion as well as components of stroma were dysregulated on the filaggrin insufficiency background, implying possible changes in cellular adhesion and migration; however, the pattern of changes did not provide sufficient indication of the direction of a possible effect.

Hence, we next followed with functional studies, i.e., adhesion assay and wound healing assay. To this end, we carried out adhesion testing using two different substrata, i.e., Matrigel (rich in laminin and collagen IV) and collagen I, with BSA used as our control. The results showed increased adhesion of the shFLG keratinocytes to the Matrigel in comparison to the shC line and an increased level of adhesion to collagen I (Fig. 5C). At the same time, the wound healing assay identified an increased speed of gap closure by the shFLG cells (Fig. 5D–E), which is in line with their increased migratory properties and the increased proliferation capacity we have previously reported [14]. Finally, we also confirmed this effect using the CRISPR-knockout N/TERT-2G line; we observed an identical effect for the ΔFLG line, which showed increased speed of the gap closure (Fig. 5F–G). This was observable already within the 24h period since the start of the assay, in line with increased proliferation rates of the knockout vs. sh knockdown keratinocytes.

## Discussion

Genetic variants in the *FLG* gene encoding a late epidermal protein, filaggrin, constitute the most prominent inherited predisposition factor for atopic dermatitis (AD) [1], highlighting the multifaceted role of this protein in supporting epidermal barrier function as well as controlling keratinocyte differentiation. Consequently, the reduction in filaggrin expression in the skin of AD patients and experimental models impacts numerous cellular and functional features during effective epidermal differentiation and cornification [11], e.g., remodeling of the cytoskeleton [12], formation of tight junctions [13], lipid content [12], and changes in the enzymatic activity in the skin [14, 15]. Pathological *FLG* variants predispose to microbial dysbiosis [22, 23] and reduced ability to control skin infections, resulting in *S. aureus* superinfections [23] and predisposition towards eczema herpeticum [39]. The impact stretches beyond the skin; *FLG* variants are also linked to the other manifestations of the “allergic march,” i.e., asthma, rhinitis, food allergy; affecting organs in which filaggrin is not expressed. Notably, inflammatory cytokines in the skin may also reduce the amount of this protein in the skin [2–10].

**Fig. 6:**
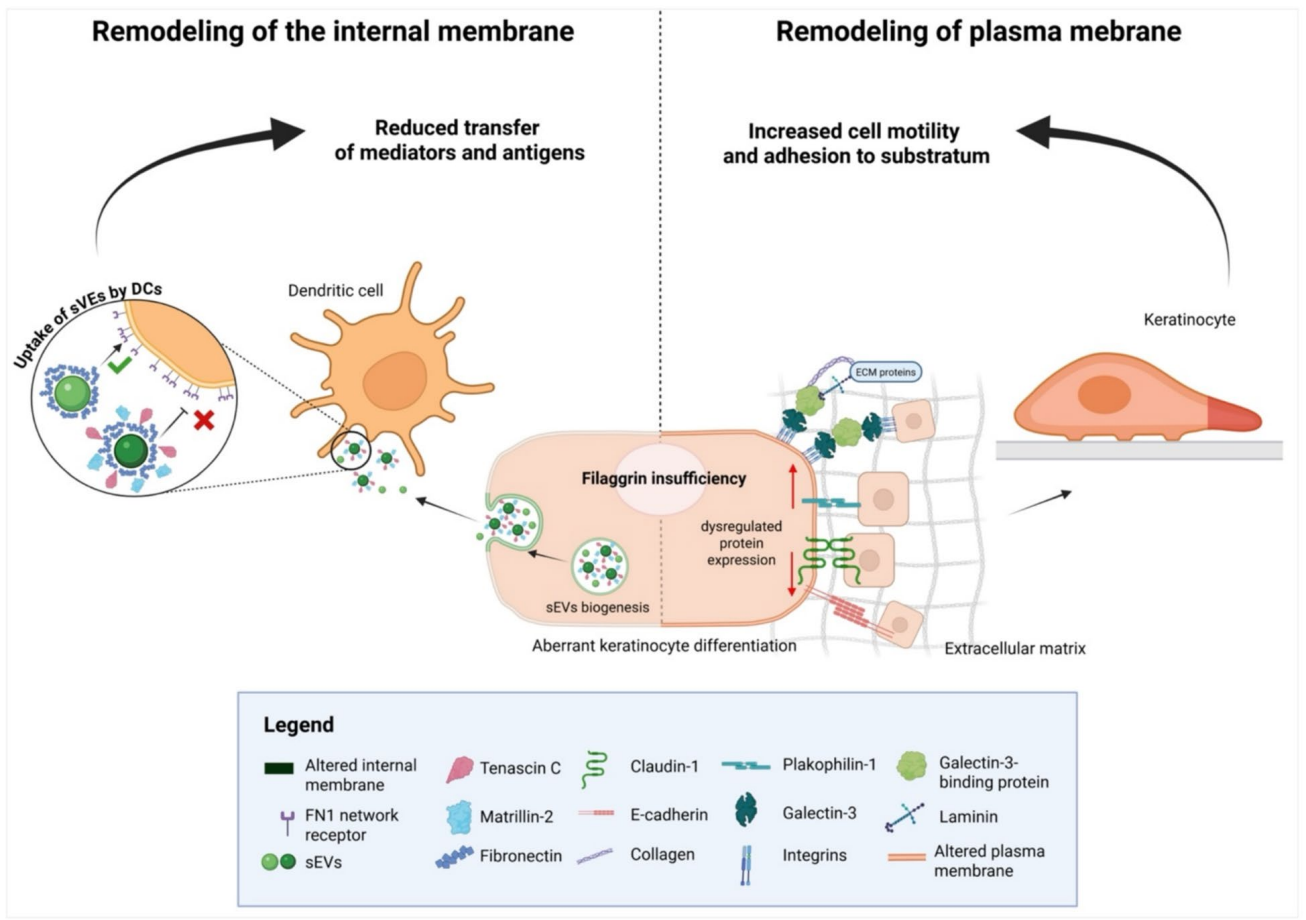
Summary of remodeling of internal and plasma membranes in filaggrin-insufficient keratinocytes

Here, we determined how filaggrin insufficiency may impact the physical membrane properties and the membrane-related cellular functionalities (Fig. 6). The experiments were carried out on keratinocyte lines since primary keratinocytes (normal human primary keratinocytes; NHEKs) are notoriously difficult to manipulate, being resistant to many methods allowing to reduce protein expression. In addition, NHEKs are also very limited in their lifespan, undergoing terminal differentiation already at passage 5–6, making it extremely difficult to select cells suitable for long-term culture, required in some of the experiments we conducted in this study, especially those requiring large cell counts, such as experiments on sEVs. These limitations were overcome with the use of keratinocyte-based filaggrin knockdown and knockout lines. Since we started this project several years ago, all the initial data in this study were obtained with the knockdown cells on the HaCaT keratinocyte background, established in the Fusenig lab in the 80s [40]; at the time of the commencement of the work, the HaCaT line was considered a suitable model for research on keratinocytes and widely used. Since then, additional lines (N/TERT) were generated by the Rheinwald lab, which have gained popularity and are now considered the gold standard, as these cells resemble primary keratinocytes more closely, especially in their ability to terminally differentiate and form a full-thickness, *stratum corneum*-including, epidermal 3D model in vitro. While we now have access to the CRISPR filaggrin knockout on the N/TERT-2G background, our resources available for this project were limited; we decided to select the functional experiments to confirm the main findings regarding both the internal membranes and plasma membrane (by the effect on the sEV uptake and wound closure, respectively). We are aware that there is a limitation that results from not being able to repeat all the experiments in the new lines; we also acknowledge that the results of the most physiologically relevant assays match the findings obtained for the HaCaT keratinocytes, giving us confidence that filaggrin insufficiency is a primary cause and the effects we observe are cell line independent. The sh- and CRISPR-based lines differ in their filaggrin reduction levels, given that the sh knockdown HaCaT lines were not selected for filaggrin downregulation and represent a mixed population, while the CRISPR line has been selected through single cell cloning. Hence, similar results obtained with both these lines signify that even in keratinocytes with a partial defect in differentiation and a lower degree of filaggrin reduction, the effect is still strong and detectable.

It is worth noting that normally the plasma membrane lipids are packed more densely than the internal organelles [26] while we could not detect a difference between the filaggrin sufficient and insufficient cells in our population-based Laurdan study; a difference was observed by holotomography. Specifically, RI measurements showed higher values in the internal membranes, indicating their higher density. To this end, polarity-sensitive dyes reflect lipid lateral packing by measuring water content around the hydrophobic fluorophore; in contrast, RI indicates general membrane condensation involving both proteins and lipids. Therefore, we hypothesize that higher intercellular condensation is likely due to an increased local protein content on the filaggrin insufficiency background and not due to the remodeling of the lipids. In fact, our results indicating the increased abundance of anti-adhesive proteins in sEVs (organelles of intracellular biogenesis), as a proxy for the internal membranes, would support this. It is important to note, however, that our study is, to our knowledge, the first ever demonstration of a cellular state which induced changes in RI of the cellular membranes detectable by holotomography, and because of this, we were not able to introduce a positive control to those experiments.

In line with the observed changes, we found that the adhesion-related quality of both the plasma membrane and membranous organelles of the intracellular origin, i.e., small extracellular vesicles (sEVs), is affected in a distinct way. Specifically, sEVs released from the filaggrin-insufficient cells showed a largely reduced propensity to be taken up by dendritic cells (DCs), regardless of the DC subset studied. Our results highlight the central role of the fibronectin-1 (FN1) network during the sEV uptake, and this is in line with the known function of FN1 during adhesion-dependent processes. For example, directional cell movement was previously demonstrated to depend on the sEV-associated FN1 included in the adhesion assembly, with importance in cancer metastasis [41]. Type 2 (i.e., pro-allergic) cytokines have been shown to reduce FN1 expression in keratinocytes, and topical application of recombinant protein was shown to improve wound healing [42].

It has been shown that both FN1 and some of the proteins found within the FN1 network may be, in fact, recovered matrix proteins that associate with the surface of the vesicles as a part of the sEV interactome rather than be incorporated into the sEV membranes [43] or contained within the sEV cargo [41] with FN1 associating with sEVs during their biogenesis in MVBs [41]. Those proteins have been previously documented to have a role in the sEV adhesion process as they form an adhesive corona on the sEV surface. It has been determined that FN1 may attach to sEV heparan sulfate [44] or integrins [45]. Integrins were not detected in sEV proteomics in our study; however, we have identified multiple proteins in the sEV samples that are known to have protein binding capacity, meaning that they could be involved in the formation of the sEV corona. Interestingly, one of the classical exosomal markers, i.e., tetraspanin CD9, which we detected, was previously also shown to interact with FN1 [46]. Interactions between EVs and FN1 specifically, and its role in the uptake of vesicles, have been also previously identified [47]. The effect we observed in this study is in line with the anti-adhesive properties of tenascin-C (TNC) and matrilin-2 (MATN2) interfering with FN1, as they are known to be involved in the modulation of adhesion [32, 48]. Specifically, while the exact role of MATN2 is not yet well understood [48, 49], it has been documented that TNC executes its function by masking FN1 adhesion domains [32], which prevents binding to cellular proteoglycans. TNC has been previously linked to the inflammatory response [50] while TNC deficiency ameliorated allergen-induced asthma in a murine model [51]. Recently, it has been also documented that TNC carried by sEVs in the bloodstream of COVID-19 patients may promote inflammation in the peripheral tissues [52]. All these studies indicate the proinflammatory function of the protein. We envisage that the reduction in the sEV adhesiveness and uptake would impact the transfer of keratinocyte-absorbed antigens, innate mediators such as pathogen-associated molecular patterns (PAMPs) or alarmin-type signals from keratinocytes in AD skin, as it has been shown previously that vesicles may contain those within their cargo [53, 54].

Our findings bear high relevance to the immunological events in the skin during microbial control [55, 56]. Epidermal keratinocytes have been shown to act as an adjuvant for immune responses in the skin [57]; the reduced uptake propensity of the sEVs secreted on the filaggrin insufficiency background may affect transfer of antigens and innate signals to Langerhans cells in the epidermis [56, 58], DC populations deeper in the tissue, or in draining lymph nodes. In consequence, less efficient pathogen clearance would promote resulting skin dysbiosis, one of the AD hallmarks. We have previously documented that keratinocyte sEVs can be hijacked by known AD pathogens, i.e., *C. albicans* [28] and *S. aureus* [59], which these pathogens could use to promote their growth on the skin. Here, a reduction of the antigenic load that the DCs may receive in sEVs could shift antigen-specific T cell response towards a tolerogenic outcome [60, 61], further contributing to the pathogen spread. Furthermore, we recently also documented that sEVs produced in this context contain lipids which promote allergic type 2 bias from antigen-specific T cells when the lipids are liberated by phospholipase A2 [25], known to be upregulated in skin disease. Hence, it is plausible that the accumulation of sEVs in the intracellular space, resulting from the reduced uptake, could also aid lipid liberation and enhance this T cell-mediated proallergic effect.

The histopathological picture in AD, encompassing hyperproliferation and spongiosis, is consistent with increased cell proliferation and reduced cell adhesion, respectively. We have previously documented [14] an increase in proliferation and a decrease in E-cadherin levels in the shFLG model [62]. In practice, both these phenomena could play a role in wound closure since single-cell migration requires loss of adherens junctions that is mediated by E-cadherin [63], suggesting a negative effect of E-cadherin on keratinocyte mobility [64]. Impaired expression of claudin-1 and dysregulated expression of additional proteins contributing to cellular adhesion could also play a role; hence, the need for functional assays in the current study to disentangle the overall effect; these confirmed the pro-healing effects. Interestingly, TNC expression is known to be upregulated in the dermis during wound healing, and in correctly healing wounds, an increased prevalence of this protein is observed at the wound edges [65, 66]. While these findings suggest that TNC supports wound healing, this process is still compromised in AD despite increased TNC presence in the skin; this could be explained by the inability of TNC to fulfill its potential role in the greater context of dysregulated extracellular matrix [ECM] remodeling occurring in this disease [67, 68].

To this end, we observed that knock-down of *FLG* triggered enhanced adhesion to both Matrigel and collagen I. Surprisingly, we observed a modest decrease in the expression of alpha1 integrin but not other components of the collagen I receptor. This suggests that *FLG* might be involved in the regulation of activity (e.g., clustering, turnover, cellular distribution) of alpha2/, alpha10/, and/or alpha11/beta1 integrins, which is reflected in increased adhesive and migratory properties of shFLG cells. Enhanced adhesion to Matrigel could result from the increased expression of galectin-3-binding protein, which interacts with laminins contained within this substratum [69]. The results obtained in this part of the study contrast those published by Dang et al. [70], who observed a reduction in migration and cellular adhesion to a similar substratum as well as a reduction in cellular proliferation. We are not aware of the reasons behind this discrepancy; however, the results they have obtained do not align with histological data in the AD skin. Since AD patients suffer from skin cracks and fissures, which are gateways for infections, mechanisms promoting fast recovery of skin integrity would be highly advantageous. However, the effect of the ongoing inflammation, with a high type 2 component [71], mechanical trauma to the skin caused by scratching, and pathogen spread, all constituting persistent clinical features in the disease, may cancel out any beneficial effect. Therefore, currently, it is difficult to assess the actual impact of our wound healing observations, especially since it does not account for the role of fibroblasts in the full-thickness skin.

## Conclusions

Our study demonstrates extensive membrane remodeling in filaggrin-deficient keratinocytes. We found that while the mechanisms driving alterations in the plasma membrane and internal membranes may vary, both appear to be mediated by membrane-associated proteins that influence adhesion-dependent processes. Additionally, we identified distinct cellular functions impacted by these changes, with implications for barrier integrity and immune cell-mediated antimicrobial defense.

## Methods

### Samples

Ethical approval for the study was obtained from the Independent Bioethics Committee for Scientific Research at the Medical University of Gdańsk (ethical approval number NKBBN/621–574/2020). Regional Blood Centre in Gdańsk provided buffy coats isolated from blood donated by healthy individuals.

### Cell culture

High-glucose Dulbecco’s modified Eagle’s medium with the addition of 10% heat-inactivated FBS, 100units/mL of penicillin, and 100μg/mL of streptomycin (all from Sigma-Aldrich) was used to culture filaggin-expressing (shC) and filaggrin-insufficient (shFLG) keratinocytes, established previously by us on a HaCaT background (HaCaT cells were a kind gift from Prof. N. Fusenig [40]). CnT-Prime Epithelial Proliferation Medium (CELLnTEC Advanced Cell System AG) was used to culture filaggrin-expressing (WT) N/TERT-2G keratinocytes, a kind gift from Prof. James Rheinwald [72], and filaggrin-insufficient (ΔFLG) N/TERT-2G keratinocytes, a kind gift from Prof. Ellen H. van den Bogaard [16]. The cells were cultured at 37°C, 5% CO2. EV-depleted FBS (centrifugation at 100,000 × g for 16h) was used to supplement cell culture media in experiments in which keratinocyte sEVs were harvested; cells were grown to 80–90% confluence for treatments.

### Flow cytometry

Cells were washed in PBS, stained with antibodies for 30 min at 4°C followed by washing in PBS, fixed in 4% formaldehyde (Sigma-Aldrich) and acquired using Guava easyCyte (Millipore). Data was analyzed with GuavaSoft 3.1.1. Antibodies were from BioLegend: CD14-APC, clone HCD14 cat. 325,608; CD40-FITC, clone 5C4, cat. 334,306; CD80-PE, clone 2D10, cat. 305,208; CD86-PE, clone IT2.2, cat. 305,438; CD1a-APC, clone HI149, cat. 300,110 at 1:20 dilution in PBS or BD Biosciences: HLA-DR-PE, clone G46-6, cat. 555,812 at 1:5 dilution in PBS.

### Western blot

RIPA buffer (Cell Signalling Technologies) with the addition of cOmplete™, Mini, EDTA-free Protease Inhibitor Cocktail (Roche) was used for cell lysis and protein extraction. Bolt™ LDS Sample Buffer (Invitrogen) was then added to cell lysates or EVs and heated for 10 min at 80°C. All samples were resolved in the Bolt™ 4–12% Bis–Tris Plus Gels (Invitrogen) using the Mini Gel Tank (Life Technologies) and the PowerEase™ 300W Power Supply (Life Technologies). Following electrophoresis, proteins were transferred onto nitrocellulose membranes (iBlot™ 2 Transfer stack; iBlot 2 Dry Blotting System, Invitrogen). The membranes were then incubated in 5% fat-removed milk in PBS with shaking for 1h followed by 3 × washing in PBS and incubating with primary antibodies diluted in PBS (CD9, clone C-4, cat. sc-13118; syntenin, clone S-31, cat. sc-100336; calnexin, clone AF18, cat. sc-23954 at 1:250 and CD63, clone MX-49.129.5, cat. sc-5275 at 1:500; Alix, clone 1A12, cat. sc-53540 at 1:200; ApoA, clone B-10, sc-376818 at 1:250; all from Santa Cruz Biotechnology) at 4 °C with shaking overnight. The membranes were washed 3 × with PBS-T (0.05% Tween 20, Sigma-Aldrich in PBS) and incubated in a secondary antibody (IRDye® 800CW Goat anti-Mouse IgG Secondary Antibody, polyclonal, cat. 926–32,212, LI-COR Biosciences) diluted in PBS-T at 1:25,000. Odyssey Clx Imaging System (LI-COR Biosciences) was used for membrane imaging.

### Monocyte-derived dendritic cells

CD14 + cells were isolated magnetically from PBMCs using MojoSort™ Human CD14 Selection Kit (BioLegend) according to the manufacturer’s protocol. Cells were grown in 24-well plates at 750,000 cells per well (Corning) in 1 ml RPMI-1640 medium (Sigma-Aldrich) supplemented with 1% Pen/Strep (Sigma-Aldrich) and 10% heat-inactivated FBS (Sigma-Aldrich) and 50 ng/ml GM-CSF and 1000 U/ml IL-4 (PeproTech). On day 2 and day 4 of the culture, the medium was replaced with fresh complete RPMI with fresh cytokines. For the generation of mMDDCs, LPS (Sigma-Aldrich) was added at 1 μg/ml on day 6. To generate tolDCs, 10 ng/ml IL-10 (PeproTech) was added on day 5, and 1 μg/ml LPS (Sigma-Aldrich) was added on day 6. Cells were harvested on day 7 for downstream analysis or experiments.

### Uptake assay and FN1 rescue assay

sEVs were density gradient-purified and labeled with the PKH67 Green Fluorescent Cell Linker Midi Kit for General Cell Membrane Labeling (Sigma-Aldrich) according to the manufacturer’s protocol. Briefly, sEVs were resuspended in Diluent C and labeled with the PKH67 Green Fluorescent Cell Linker Kit (Sigma-Aldrich); for a mock control, Diluent C alone was used for labeling. The labeling reaction was quenched by the addition of 2 × volume of RPMI medium (Sigma-Aldrich) supplemented with 10% EV-depleted FBS. Samples were then washed in PBS at 100,000 × g for 19 h at 4 °C, and sEVs were resuspended in EV-depleted complete RPMI medium. MDDCs or THP-1 cells were pulsed with the labeled sEVs and incubated for 4 h at 37 °C, 5% CO_2_. An equivalent of 10 µg/ml of sEVs by protein concentration of intact sEVs, as measured using NanoDrop 2000 (Thermo Fisher Scientific), was added to cells for pulsing. As a control, 40kDa dextran-FITC (Chondrex) was added to the cell culture at 0.1 mg/ml for pulsing.

For the rescue assay experiment, labeled sEVs were pre-incubated with 5 or 50 µg/mL recombinant FN1 (R&D Systems) for 30 min on ice. Samples were then washed in PBS at 100,000 × g for 19 h, at 4 °C, and sEVs were resuspended in EV-depleted complete RPMI medium. THP-1 cells were washed twice with PBS and pulsed with either PKH67-labeled shC-, shFLG-derived sEVs, or mock control, and incubated for 4 h at 37 °C. Cells were then washed, fixed in 4% formaldehyde (Sigma-Aldrich), and analyzed using the Millipore Guava EasyCyte Flow Cytometer (Merck Millipore) or by Tomocube HT-2H microscope for 3D fluorescent holotomography reconstruction (RI-based digital staining) with green channel acquisition for the assessment of the dextran or labeled sEVs.

### Holotomography and fluorescence imaging

Holotomographic and fluorescent images were obtained using a Tomocube HT-2H (Tomocube) microscope. For holotomographic imaging, a monochromatic laser (λ = 532 nm) was used, allowing RI (refractive index)-based image acquisition. A 1.34–1.36 RI value range was used to visualize the plasma membrane and 1.37–1.42 to image internal membranes. For fluorescence imaging, a LED light source (λ = 470 nm) was used. Serial fluorescent image acquisition in multiple planes was performed to reconstruct the signal in 3D. Visualization of 3D images was carried out using Tomocube dedicated software (TomostudioTM, Tomocube). N.B.: since our study is, to our knowledge, the first study using holotomography specifically for the assessment of changes in the membrane RI, a suitable control (e.g., a compound which is known to induce RI changes in the membranes by this technique) was not available to serve as a positive control in this case.

### GP assessment with Laurdan

shC and shFLG cells were seeded on a 96-well tissue culture-treated flat-bottom plate. Upon reaching confluence, cells were treated with 5 µl of 1 mM Laurdan (6-dodecanoyl-2-dimethylamino naphthalene; Sigma-Aldrich), reconstituted in DMSO for 10 min at room temperature in the dark. Afterwards, cells were washed twice with PBS and the plate was read on the EnVision™ Multimode Plate Reader (PerkinElmer) (excitation wavelength at 380 nm, emission wavelength at 440 nm and 490 nm); each plate was read 10 times for a total of 5 biological repeats. Calculations of the GP values were done as published before [26].

### EV isolation, purification, and characterization

Media supplemented with EV-free FBS was used in experiments. Conditioned media was collected 72 h after cell treatment and subjected to differential centrifugation (all steps at 4 °C); first at 300 × g for 10 min (Megafuge 16R TH-400, Thermo Scientific) to remove cells and debris, then at 2000 × g for 10 min to eliminate insoluble proteins and apoptotic bodies followed by centrifugation at 10,000 × g for 30 min (OptimaTM L-90 K or OptimaTM LE-80 K, Beckman Coulter) to remove microvesicles and finally exosome-enriched sEVs were pelleted at 100,000 × g for 16–19 h. For HaCaT-derived sEVs, density gradient purification was performed; sEVs were top-loaded onto an iodixanol/sucrose gradient cushion (6–18% iodixanol with 1.2% difference between adjacent cushion layers; 1 ml per layer) and centrifuged in a swinging-bucket rotor at 142,000 × g (AVG) for 2.5 h at 4 °C (SW 41 Ti, Beckman Coulter). One-milliliter fractions were collected (1st fraction was considered the top layer (1 ml) + top-loaded sample volume) and fractions 1–5, which contained purified exosome-enriched sEVs, were pooled. Collected fractions were washed in PBS at 100,000 × g for 16–19 h at 4 °C.

Number and size of sEVs were measured by Nanoparticle Tracking analysis (NTA) with NanoSight NS300 fitted with a 488 nm laser (Malvern Panalytical). 3 recordings were acquired per sample, 30 s each. Electron microscopy images were acquired by the Laboratory of Electron Microscopy at the University of Gdańsk and provided as a paid service; sEVs were transferred onto formvar/carbon film-coated copper grids (300 mesh) (EM Resolutions) followed by negative staining with 1.5% uranyl acetate (BD Chemicals Ltd.). sEV images were then recorded using the Tecnai G2 Spirit BioTWIN (FEI Inc.) transmission electron microscope.

### Adhesion assay

shC and shFLG cells were seeded into fresh 60-mm cell culture dishes the day before an assay. A 96-well plate was coated with 200 μg/ml of freshly prepared Matrigel® Basement Membrane Matrix or rat tail Collagen type I overnight at 4 °C. Coating with 200 μg/ml of BSA (Carl Roth) was used as a control. Next, cells were detached with enzyme-free cell dissociation buffer EDTA-based (Millipore), washed, resuspended in serum-free medium, and seeded into pre-coated wells (5 × 10^4^ cells per well) in triplicates. Cells were allowed to attach for 60 min at 37ºC and stained with 10 μM 2′,7′-Bis- (2-Carboxyethyl)−5-(and-6)-Carboxyfluorescein, Acetoxymethyl Ester (BCECF, AM; ThermoFisher) for 15 min at RT, protected from light. After 3–5 washing steps with PBS, the fluorescence signal of the attached cells was measured using a microplate reader (excitation wavelength at 439 nm, emission wavelength at 535 nm).

### Wound healing assay

The cells were grown until confluent, and the scratches were made in the cell monolayer with a pipette tip. The cells were washed twice with PBS, and culture medium was added. For the shFLG and shC cells, images were captured every 6 h for HaCaTs and every 2 h for N/TERT-2G. Wound closure is represented as the % of wound closure at 24, 48, and 72h. For the WT and ΔFLG N/TERT-2G cells, which grow faster in tissue culture, images were captured at 2, 4, 8, and 24h time points.

### Protein mass spectrometry

sEVs were purified by density gradient centrifugation to remove possible contamination of keratohyalin granules or large protein aggregates. Keratinocytes and sEVs were lysed using 1% SDS. Samples were further processed for the mass spectrometry analysis by following the Filter Aided Sample Preparation (FASP) procedure [73] with cysteine alkylation by iodoacetamide and proteolytic digestion by trypsin. Digested samples were then desalted according to the STAGE Tips [74] procedure on a C18 resin. LC–MS/MS was performed on a Triple TOF 5600 + mass spectrometer (SCIEX) coupled with Ekspert MicroLC 200 Plus System (Eksigent). All samples were measured in the data-dependent acquisition mode for the spectral library construction by the SWATH-MS [75] method in triplicate for relative quantification. Separate spectral libraries for the cell and sEV samples were constructed via ProteinPilot 4.5 software (SCIEX) against the SwissProt Homo sapiens database updated on 02.07.2020. SWATH-MS measurements were processed with respective libraries in the PeakView 2.2 software. Resulting protein intensities were normalized by total area sums (TAS) approach and imported into the Perseus software [76], where the technical replicates were median-averaged, and the resulting values were log2-transformed and normalized by z-score. Statistical significance of differential protein expression was determined by *t*-test between the test and control groups, and *p*-values were FDR-adjusted; values lower than 0.05 were considered statistically significant. The proteomic data analyzed in this study had been previously generated in relation to a separate project and deposited by us to the ProteomeXchange Consortium via the PRIDE partner repository and were assigned the following identifier: PXD026859 [25, 38, 77].

### Functional enrichment, Gene Ontology, and molecular network analysis

Cellular compartment enrichment analysis of the omics datasets was performed using FunRich 3.1.3 software. The Vesiclepedia [78] database available within the software was used to investigate the association of proteins/gene products identified in the omics studies with exosomes. Gene Ontology (GO) and Reactome pathways analysis were carried out via the Gone Ontology tool, available at http://geneontology.org/. Complete GO annotation datasets were chosen. For the GO network visualization, we performed a semantic similarity analysis of GO terms using GOATOOLS [79] to construct a network of related biological processes. Nodes and edges representing the GO terms and their relationships were imported into Cytoscape v. 3.8.2 software (https://cytoscape.org/) for visualization, including isolated nodes for a comprehensive overview. Interactions between the proteins of interest were identified using the STRING database [80] available in Cytoscape via the stringApp. NB. trypsin was considered a contaminant of the MS sample processing and removed from the network for the purpose of the analysis.

### Analysis of protein–protein interactions

The 3D structures of the proteins were obtained from the AlphaFold Protein Structure Database (AlphaFold DB), with the respective AlphaFoldDB IDs [P02751, P24821, O00339]. AlphaFoldDB (https://alphafold.ebi.ac.uk) is a publicly accessible database containing highly accurate predictions for over 200 million protein structures [81]. Protein–protein docking was performed using the ClusPro server (version 2.0), following the default settings provided by ClusPro [82]. Binding affinities of the docked protein–protein complexes were calculated using the PRODIGY web server (https://rascar.science.uu.nl/new/prodigy). PRODIGY is a collection of web services designed to predict binding affinity in biological complexes and to identify biological interfaces from crystallographic data [83]. The docking results were subsequently analyzed and visualized using UCSF ChimeraX [84], developed by the Resource for Biocomputing, Visualization, and Informatics at the University of California, San Francisco, with support from the National Institutes of Health R01-GM129325 and the Office of Cyber Infrastructure and Computational Biology, National Institute of Allergy and Infectious Diseases.

### mRNA microarray and publicly available transcriptomic dataset analysis

Upon reaching 80% confluence, shC and shFLG keratinocytes were stimulated for 24h with cytokines (IFNy or a cocktail of IL-4/IL-13) (Peprotech) at 50ng/ml each. This was followed by RNA isolation from the cells using the RNeasy kit (Qiagen) by following the instructions provided with the kit. RNA was sent out for microarray analysis to ServiceXS; Illumina HT12v4 BeadArray platform (Illumina) was used by the company. Normalization and analysis of data were performed according to lumiref and LIMMAref, respectively. The data analyzed in this study had been previously generated in relation to a separate project and deposited by us into the Gene Expression Omnibus repository under accession number GSE203409.

Skin transcriptomic datasets available via GEO2R under accession numbers: GSE16161 [11, 85], GSE6012 [86, 87], GSE107361 [88, 89], GSE130588 [90, 91], and GSE32924 [92, 93] were analyzed for TNC and MATN2 expression using default settings and log2FC of mRNA expression in lesional AD vs healthy control skin, and FDR-adjusted *p*-values were computed. TNC and MATN2 mRNA expression in non-lesional AD vs healthy control skin was extracted from datasets published in Cole et al. as Tables E4 and E5 [31].

### Statistical analysis

The Student’s *t* test, one-way and two-way analysis of variance (ANOVA), Kruskal–Wallis tests, with multiple comparison corrections were performed using GraphPad Prism v.7.04 or newer (GraphPad Software). Error bars represent SEM as indicated.

## Supplementary Information

Additional file 1. Fig. S1. Assessment of keratinocyte membrane lipid packing by Laurdan staining; pooled data from n=5 biological replicates; unpaired t-test; ns.Fig. S2. Example size profiles of shC and shFLG sEVs by Nanoparticle Tracking Analysis (NTA).Fig. S3. **A-B** Venn diagrams showing the overlap between keratinocyte-derived sEV proteins (pink circle) and **A** differentially expressed proteins by shFLG keratinocytes (blue circle) or **B** all keratinocyte proteins detected by mass spectrometry (blue circle). **C** Venn diagram depicting an overlap between Vesiclepedia-identified exosomal proteins (blue area) and differentially expressed keratinocyte proteins detected by mass spectrometry; all differentially expressed proteins (upregulated or downregulated) in shFLG cells in pink area, downregulated proteins in shFLG cells in orange field, upregulated proteins in shFLG cells in yellow area. **D** STRING protein-protein interaction analysis of all keratinocyte sEV proteins detected by mass spectrometry in shC and shFLG cells. **E** STRING protein-protein interaction analysis of keratinocyte sEV proteins linked to the extracellular matrix (ECM).**F** STRING analysis showing TNC and MATN2 interaction network.Fig. S4. STRING protein-protein interaction analysis of top 50 differentially expressed proteins by shFLG keratinocytes identified by mass spectrometry.Fig. S5. Binding of FN1 (purple) to both TNC (pink) and MATN2 (green) visualized by the ChimeraX molecular visualization program; left: ribbon view; right: surface models.Fig. S6. **A-B** Activation marker expression by MDDCs measured by FACS; **A** example plots; MFI is shown; **B** combined MFI data from *n *= 6 donors; means with SEM are shown; one-way ANOVA with Šídák’s multiple comparisons test; **p<0.01, ***p<0.001, ****p<0.0001.**C-E** FITC-conjugated dextran uptake by MDDCs measured by FACS; **C** example plots; % positive cells (upper values) and MFI (lower values) are shown; combined **D** % positive cells and **E** MFI data from *n *= 9 donors; means with SEM are shown; one-way ANOVA with Tukey’s multiple comparisons test;**p<0.01, ****p<0.0001. **F** 3D-Holotomographic reconstruction of dendritic cell models pulsed with PKH67-labeled N/TERT-2G WT and ΔFLG keratinocyte-derived sEVs.Fig. S7. **A** Characterization of the sEVs secreted by FLG WT and ΔFLG N/TERT-2G; WB and NTA. **B** Example FACS plots of the uptake of PKH67-labeled sEVs produced by N/TERT-2G WT and ΔFLG keratinocytes by THP-1 cells.

Additional file 2. Table S1: List of Gene Ontology terms related to internal cellular membrane compartments remodeling; terms were extracted from the previously published [25] Gene Ontology analysis of differentially expressed mRNA/proteins in various filaggrin insufficiency models. 

Additional file 3. Table S2: List of proteins detected in shC and shFLG sEVs.

Additional file 4.Uncropped WB membranes.

Additional file 5. Movie S1: Rotational video providing a comprehensive 360-degree view of the interaction between FN1 (purple) and TNC (pink) in a ribbon representation visualized by the ChimeraX program.

Additional file 6. Movie S2: Rotational video providing a comprehensive 360-degree view of the interaction between FN1 (purple) and TNC (pink) in a surface representation visualized by the ChimeraX program.

Additional file 7. Movie S3: Rotational video providing a comprehensive 360-degree view of the interaction between FN1 (purple) and MATN2 (green) in a ribbon representation visualized by the ChimeraX program.

Additional file 8. Movie S4: Rotational video providing a comprehensive 360-degree view of the interaction between FN1 (purple) and MATN2 (green) in a surface representation visualized by the ChimeraX program.

Additional file 9. Movie S5: Rotational video providing a comprehensive 360-degree view of FN1 (purple) interacting with TNC (pink) and MATN2 (green) in a ribbon representation visualized by the ChimeraX program.

Additional file 10. Movie S6: Rotational video providing a comprehensive 360-degree view of FN1 (purple) interacting with TNC (pink) and MATN2 (green) in a surface representation visualized by the ChimeraX program.

## Data Availability

shC and shFLG keratinocyte and sEV proteomic data have been deposited to the ProteomeXchange Consortium via the PRIDE partner repository and were assigned the following identifier: PXD026859 [25, 38, 77]. shC and shFLG keratinocyte mRNA microarray data were deposited into the Gene Expression Omnibus repository, under accession number GSE203409 [25, 28, 37]. Skin transcriptomic datasets are available via GEO2R under accession numbers: GSE16161 [11, 85], GSE6012 [86, 87], GSE107361 [88, 89], GSE130588 [90, 91], and GSE32924 [92, 93]. The RNA-seq dataset of Cole et al. [31] is available as Tables E4 and E5 in that article.

## Abbreviations

AD: Atopic dermatitis
sEV: Small extracellular vesicle
GO: Gene Ontology
shFLG: Short hairpin RNA-mediated FLG knockdown
shC: Filaggrin-expressing (short hairpin RNA knockdown control)
shC_sEV_: Small extracellular vesicle produced by filaggrin-expressing HaCaT
shFLG_sEV_: Small extracellular vesicle produced by filaggrin-knockdown HaCaT
GP: General polarization
RI: Refractive index
TEM: Transmission electron microscopy
NTA: Nanoparticle tracking analysis
WB: Western blot
EV: Extracellular vesicle
HSPA8: Heat-shock protein 70 protein 8
LGALS3BP: Galectin-3-binding protein
FN1: Fibronectin-1
TNC: Tenascin-C
MATN2: Matrilin-2
Kd: Dissociation constant
iMDDC: Immature monocyte-derived dendritic cell
mMDDC: Mature monocyte-derived dendritic cell
tolDC: Tolerogenic dendritic cell
ApoA: Apolipoprotein A
DC: Dendritic cell
BSA: Bovine serum albumin
ECM: Extracellular matrix
PAMP: Pathogen-associated molecular pattern
RIPA: Radioimmunoprecipitation assay
PBMC: Peripheral blood mononuclear cell
LPS: Lipopolysaccharide
FASP: Filter Aided Sample Preparation
STAGE: STop And Go Extraction
LC–MS/MS: Liquid chromatography-tandem mass spectrometry
TOF: Time-of-flight
SWATH-MS: Sequential Window Acquisition of all Theoretical fragment ions Mass Spectrometry
