# Supplementary material for: Remodeling of the cell membrane-associated protein pool affects adhesive membrane properties in filaggrin insufficient keratinocytes and impacts distinct cellular and organellar functions
Source: BMC Biol. 2026 Jan 8;24:30. doi: 10.1186/s12915-025-02499-y (PMC12874910; doi:10.1186/s12915-025-02499-y)
Supplement: Supplementary file 1 — Additional file 1. Fig. S1. Assessment of keratinocyte membrane lipid packing by Laurdan staining; pooled data from n=5 biological replicates; unpaired t-test; ns.Fig. S2. Example size profiles of shC and shFLG sEVs by Nanoparticle Tracking Analysis (NTA).Fig. S3. A-B Venn diagrams showing the overlap between keratinocyte-derived sEV proteins (pink circle) and A differentially expressed proteins by shFLG keratinocytes (blue circle) or B all keratinocyte proteins detected by mass spectrometry (blue circle). C Venn diagram depicting an overlap between Vesiclepedia-identified exosomal proteins (blue area) and differentially expressed keratinocyte proteins detected by mass spectrometry; all differentially expressed proteins (upregulated or downregulated) in shFLG cells in pink area, downregulated proteins in shFLG cells in orange field, upregulated proteins in shFLG cells in yellow area. D STRING protein-protein interaction analysis of all keratinocyte sEV proteins detected by mass spectrometry in shC and shFLG cells. E STRING protein-protein interaction analysis of keratinocyte sEV proteins linked to the extracellular matrix (ECM).F STRING analysis showing TNC and MATN2 interaction network.Fig. S4. STRING protein-protein interaction analysis of top 50 differentially expressed proteins by shFLG keratinocytes identified by mass spectrometry.Fig. S5. Binding of FN1 (purple) to both TNC (pink) and MATN2 (green) visualized by the ChimeraX molecular visualization program; left: ribbon view; right: surface models.Fig. S6. A-B Activation marker expression by MDDCs measured by FACS; A example plots; MFI is shown; B combined MFI data from n = 6 donors; means with SEM are shown; one-way ANOVA with Šídák’s multiple comparisons test; **p<0.01, ***p<0.001, ****p<0.0001.C-E FITC-conjugated dextran uptake by MDDCs measured by FACS; C example plots; % positive cells (upper values) and MFI (lower values) are shown; combined D % positive cells and E MFI data from n = 9 donors; means with [file 12915_2025_2499_MOESM1_ESM.docx]

**Additional file 1: Figures S1-S7.**

**
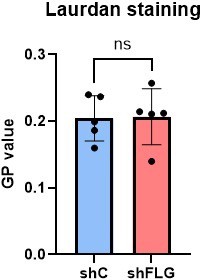
**

**
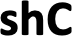

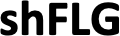
**

**
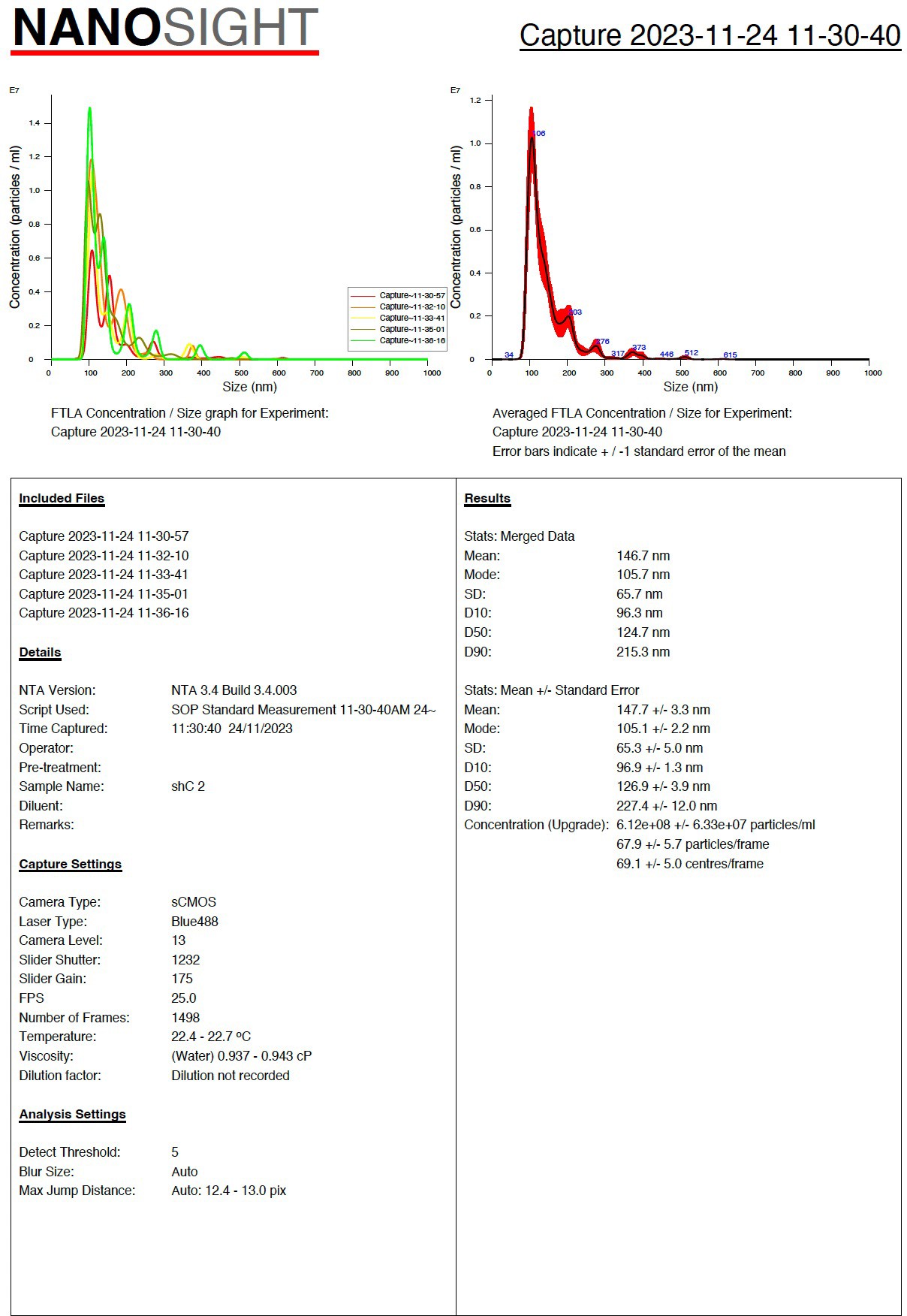

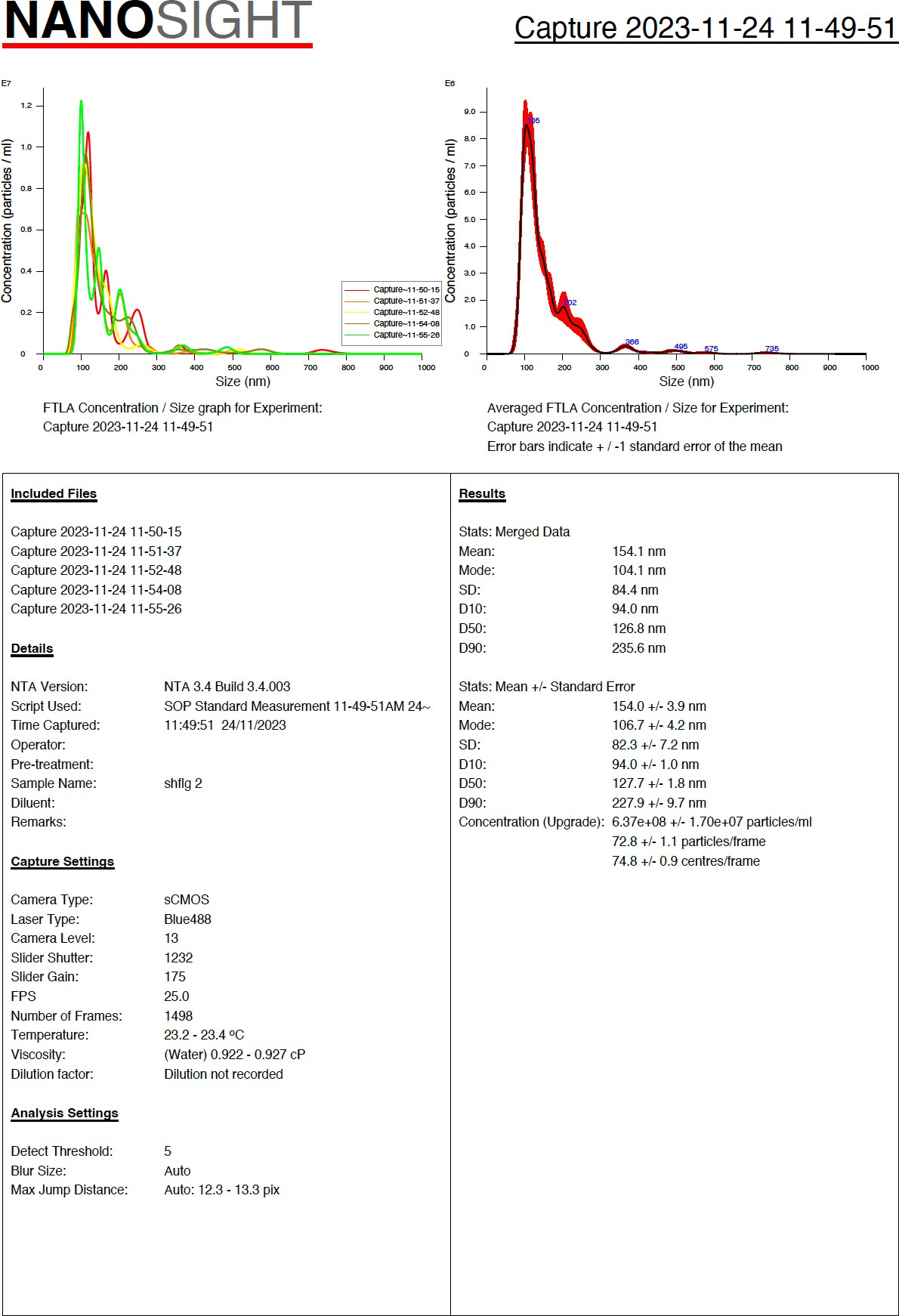
**

##
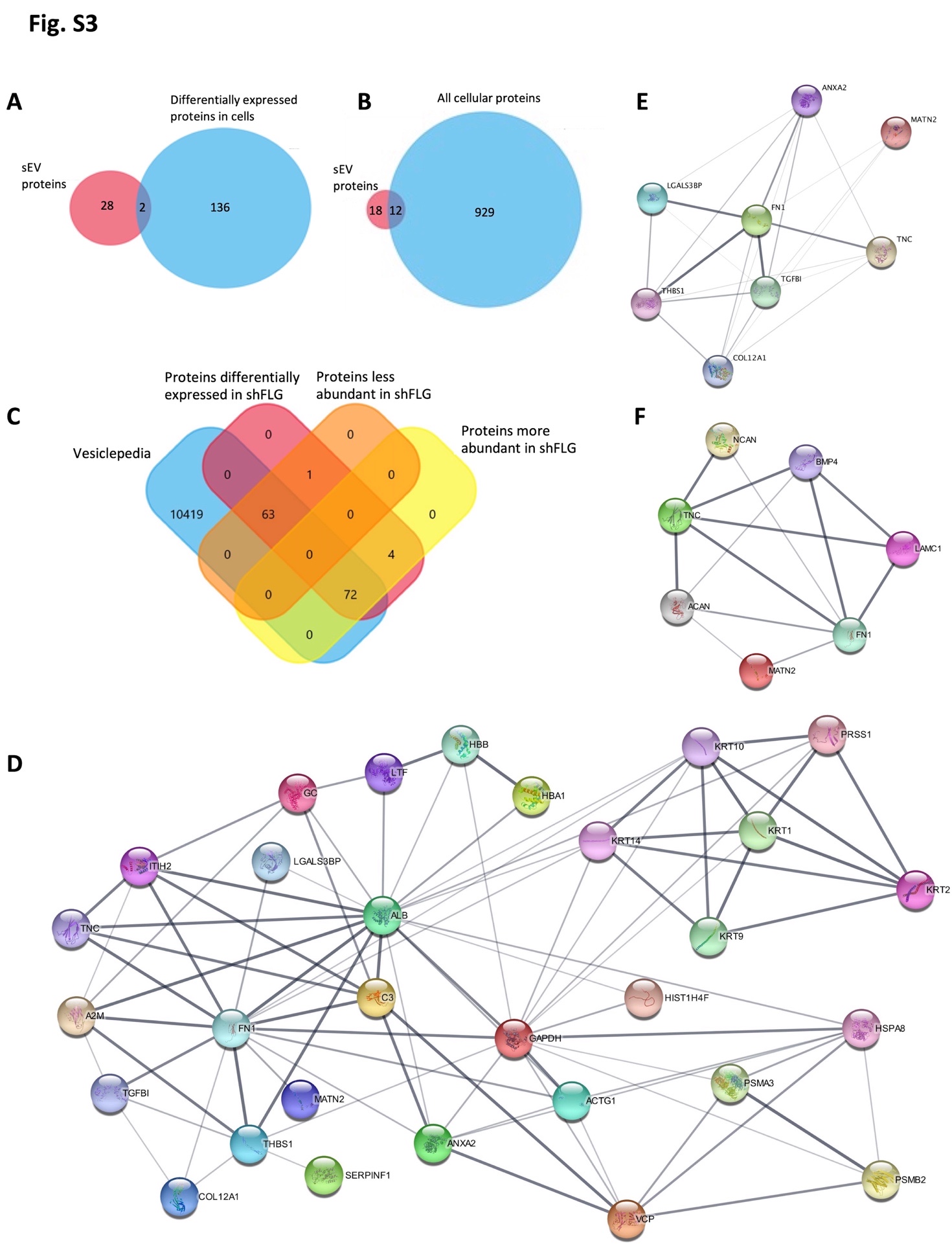


**
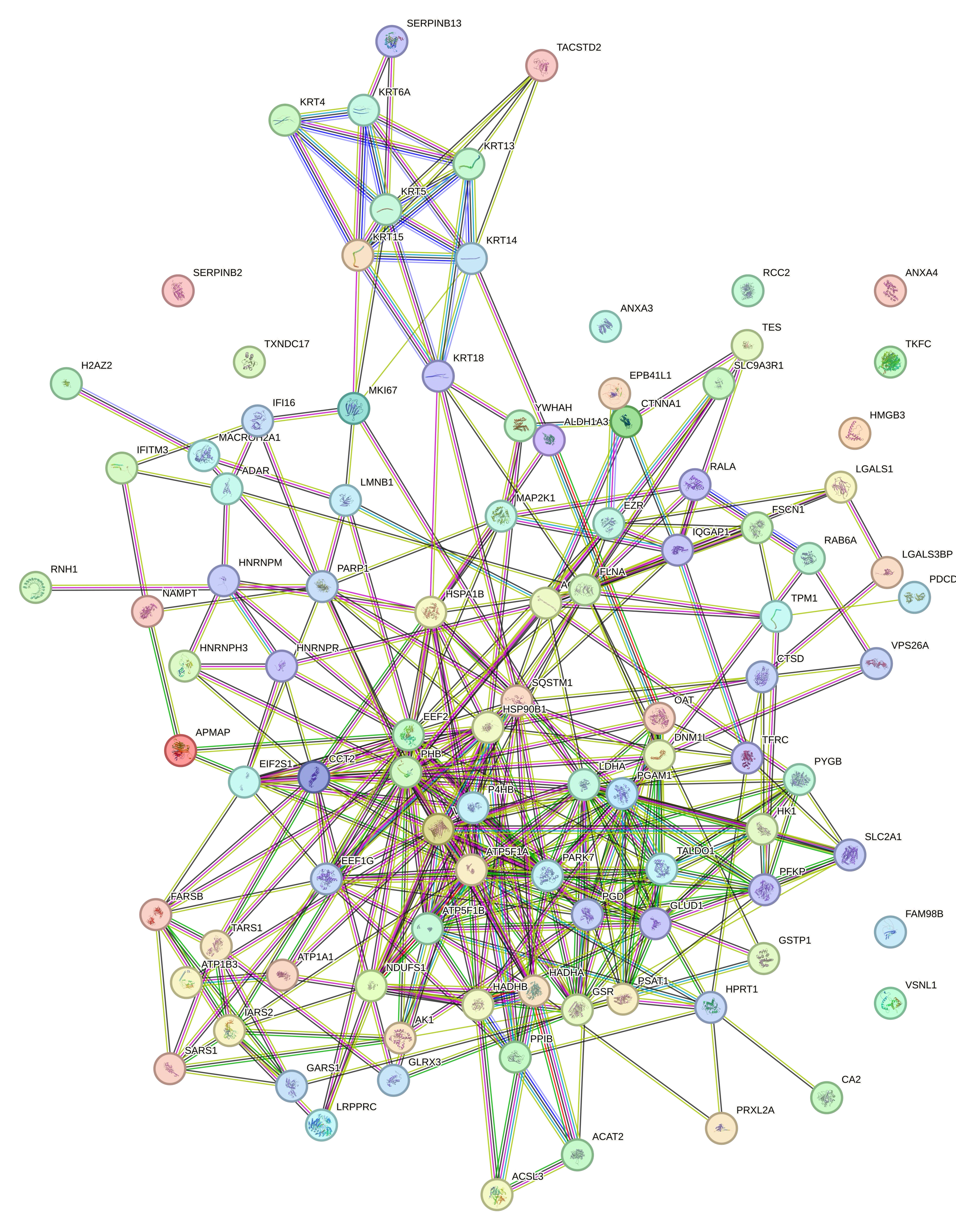
**


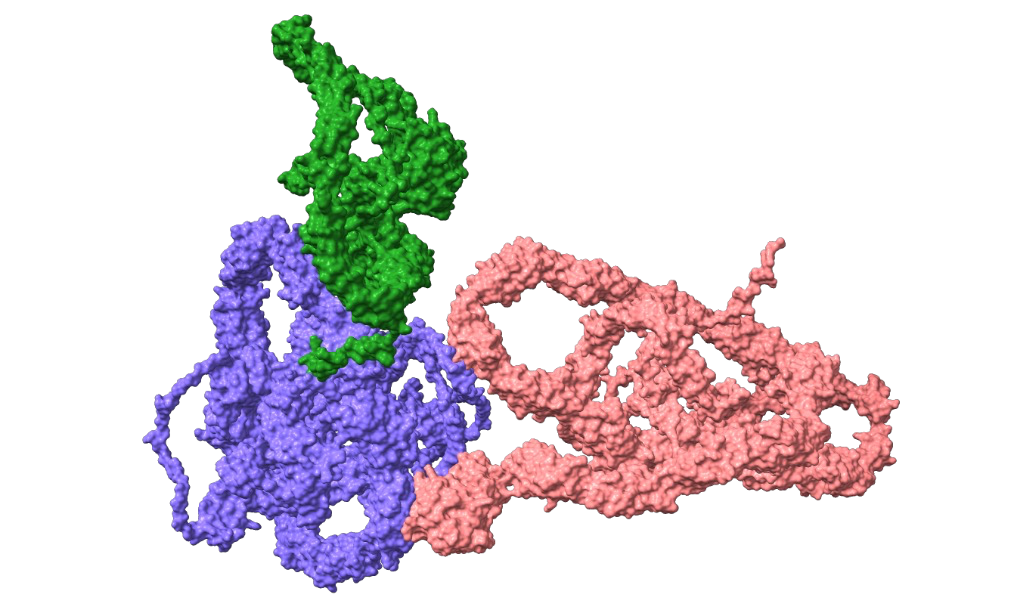

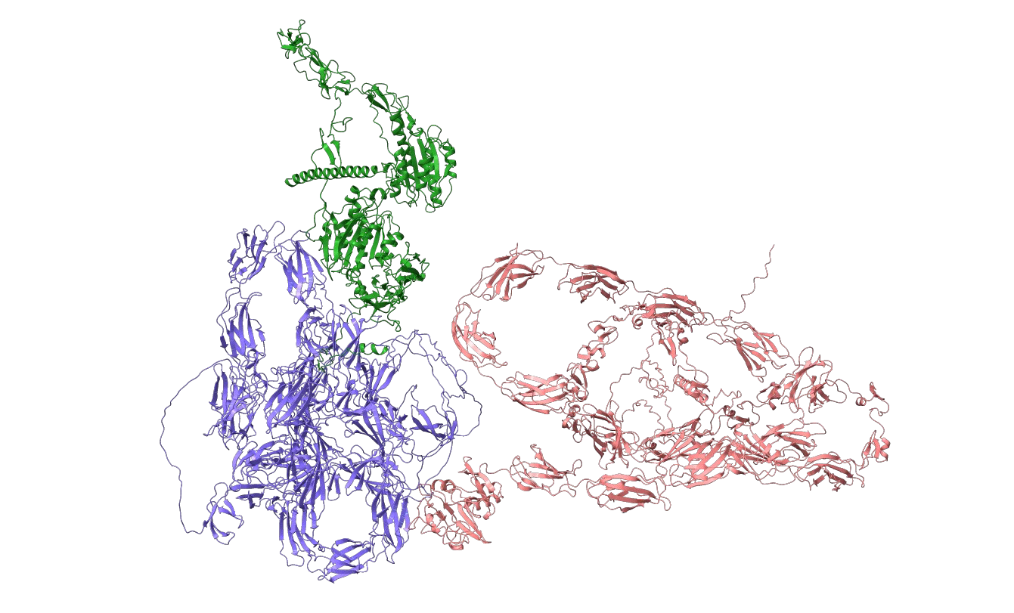


**
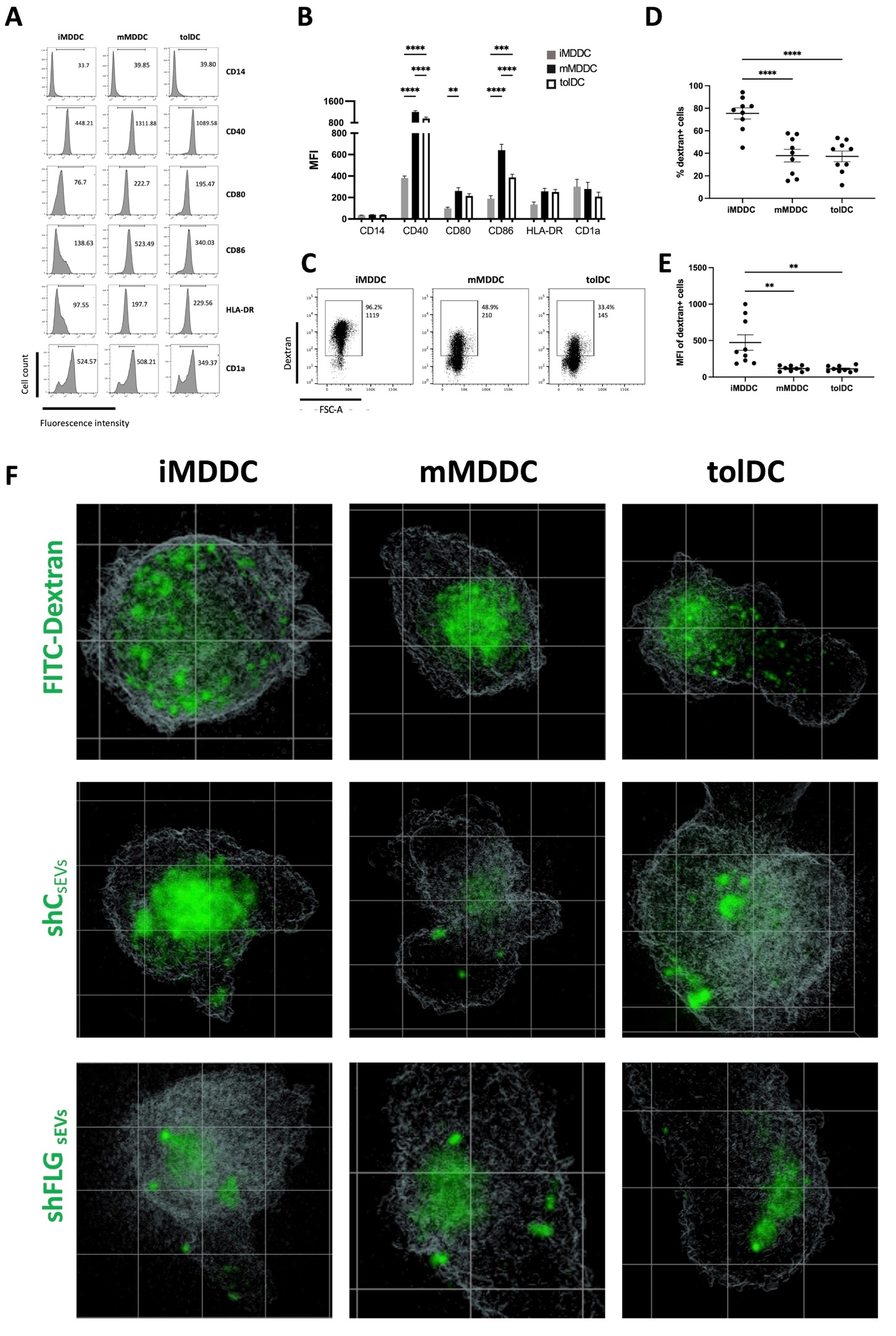
**

## A

ΔFLG sEVs

WT sEVs

ΔFLG lysate

WT lysate

ΔFLG sEVs

WT sEVs

ΔFLG lysate

WT lysate

**
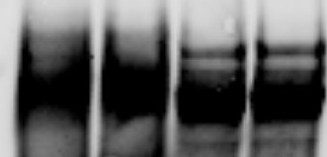
CD63**

**
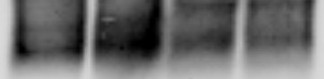
 CD9**

**
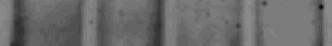
 Alix**

**
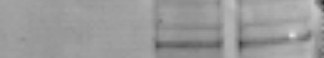
 Calnexin
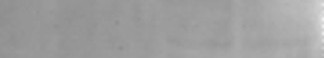
 ApoA**

# WT ΔFLG

**
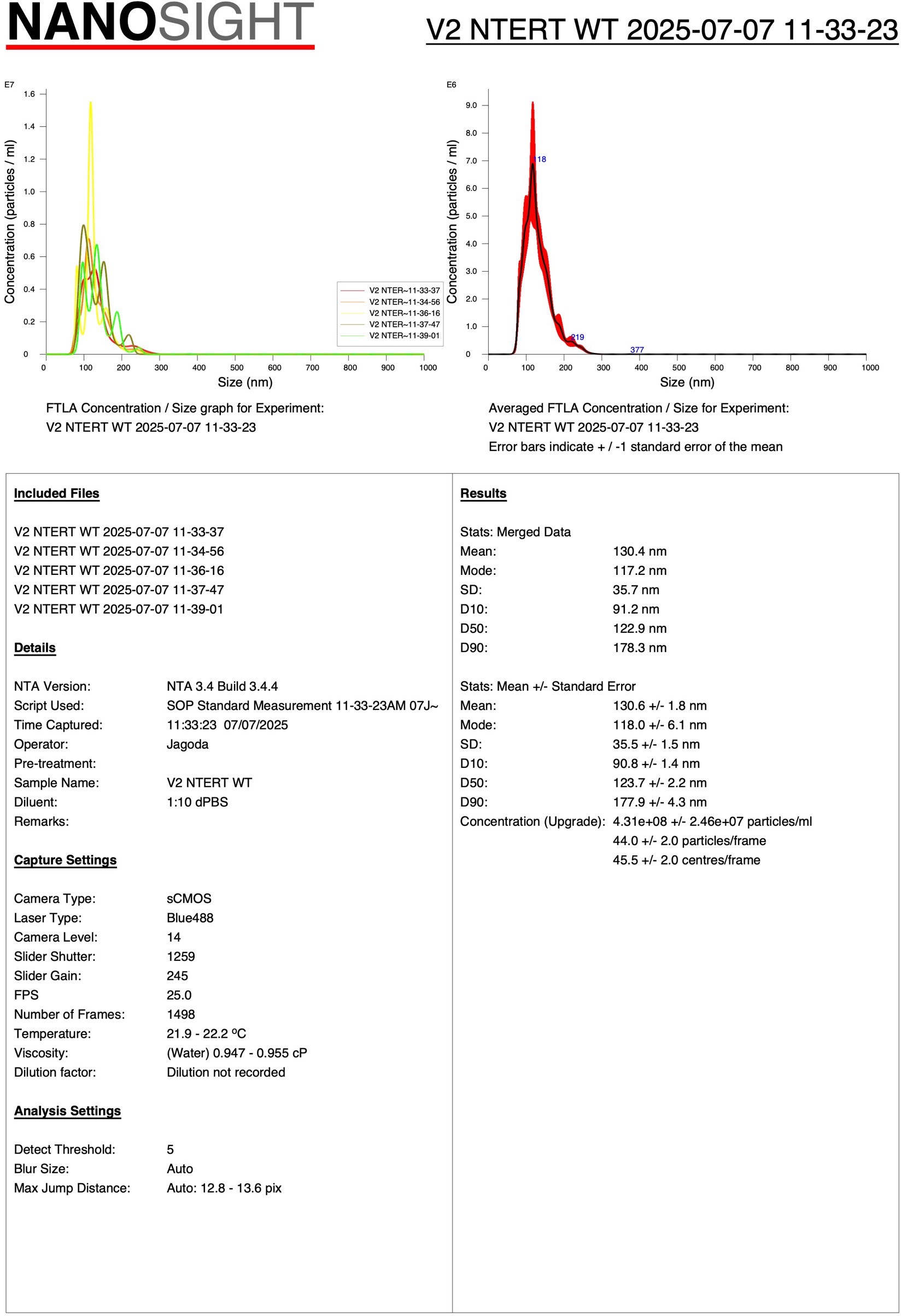

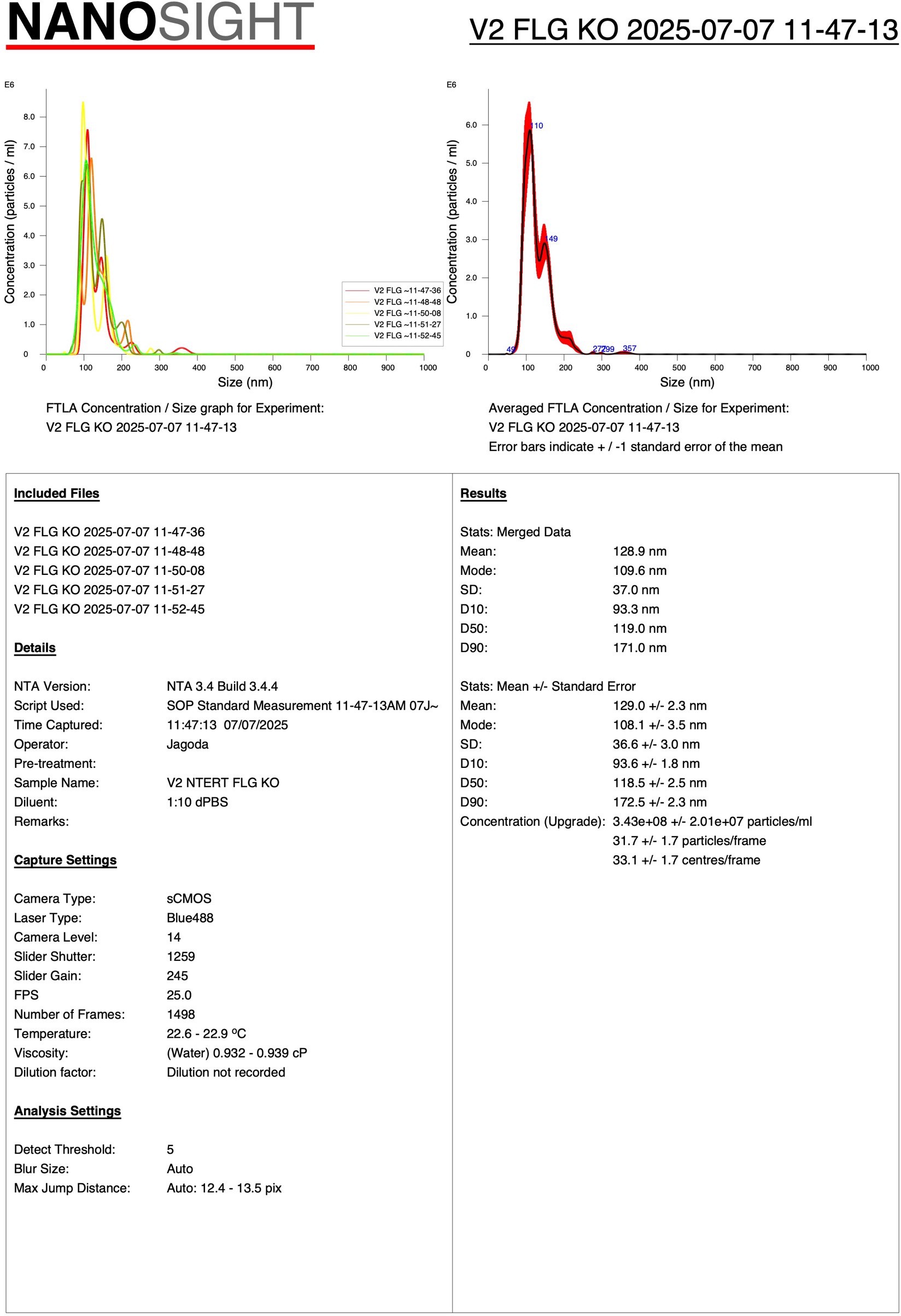
**

**
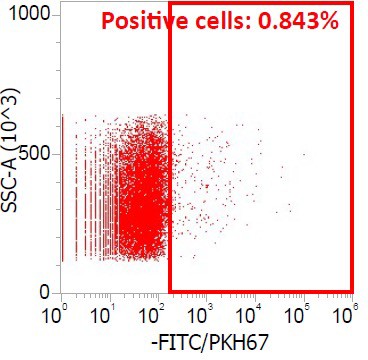

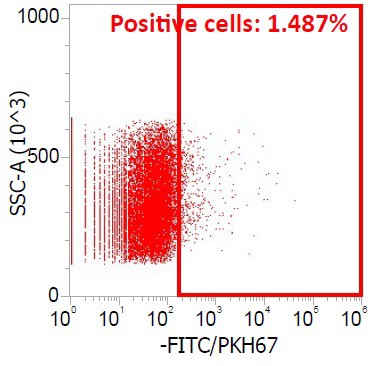

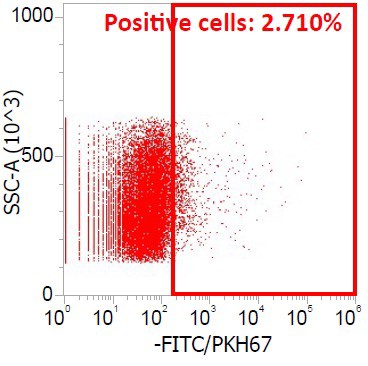
B Mock WT FLG KO**
