# Supplementary figures and images for: Remodeling of the cell membrane-associated protein pool affects adhesive membrane properties in filaggrin insufficient keratinocytes and impacts distinct cellular and organellar functions
Source: BMC Biol. 2026 Jan 8;24:30. doi: 10.1186/s12915-025-02499-y (PMC12874910; doi:10.1186/s12915-025-02499-y)

Uncropped WB membranes  
from Figure 2

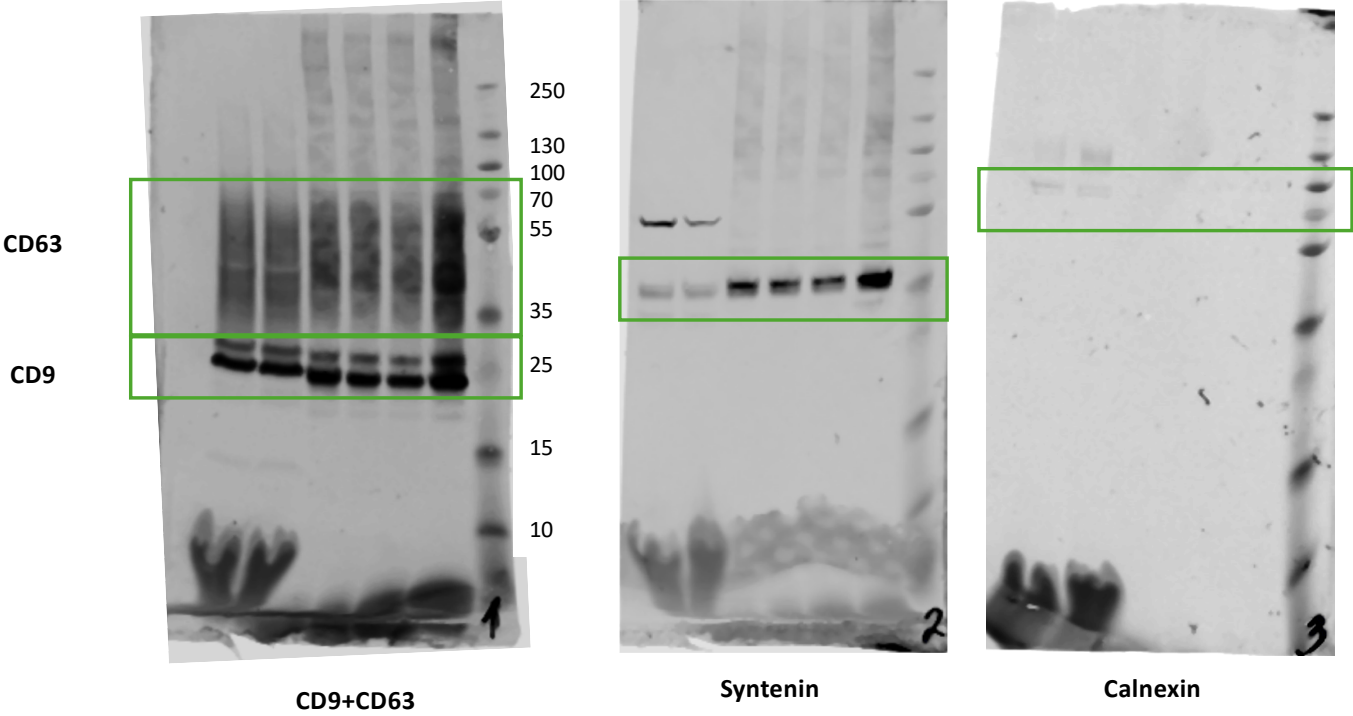

**Uncropped WB membranes  
from figure S7**

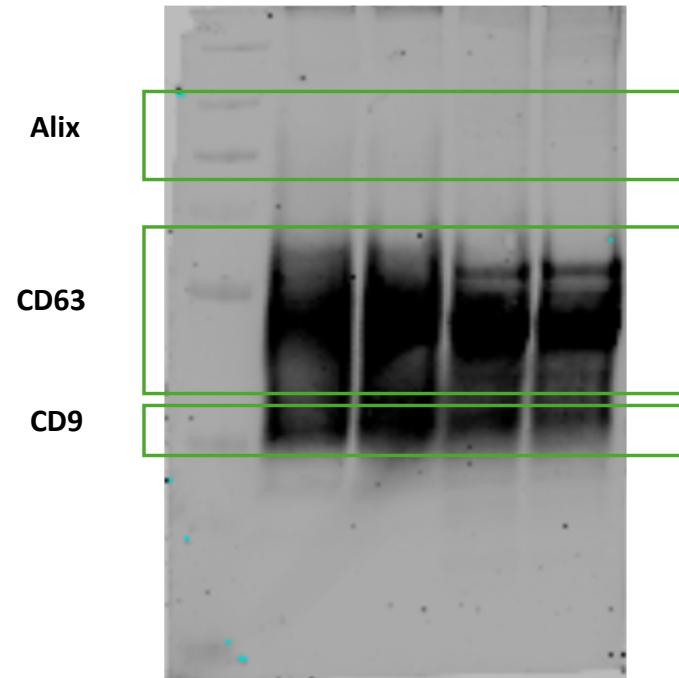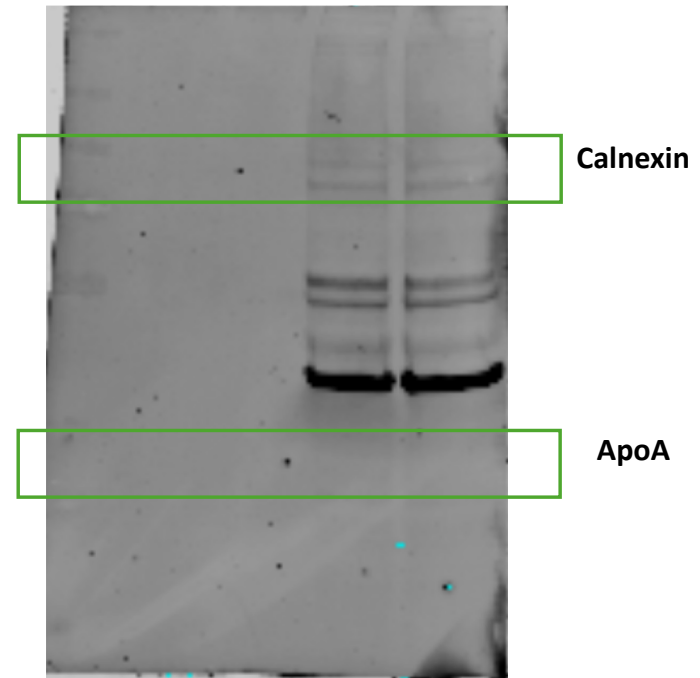

Supplement: Supplementary file 4 — Additional file 4.Uncropped WB membranes. [file 12915_2025_2499_MOESM4_ESM.pdf]
